# Rebalancing gene haploinsufficiency *in vivo* by targeting chromatin

**DOI:** 10.1038/ncomms11688

**Published:** 2016-06-03

**Authors:** Filomena Gabriella Fulcoli, Monica Franzese, Xiangyang Liu, Zhen Zhang, Claudia Angelini, Antonio Baldini

**Affiliations:** 1 CNR Institute of Genetics and Biophysics Adriano Buzzati Traverso, Via Pietro Castellino 111, Naples 80131, Italy; 2 Istituto per le Applicazioni del Calcolo, CNR, Naples, Italy; 3 Shanghai Pediatric Congenital Heart Institute, Institute for Pediatric Translational Medicine, Shanghai Children's Medical Center, Shanghai Jiaotong University School of Medicine, Shanghai 200127, China; 4 Department of Molecular Medicine and Medical Biotechnology, University of Naples Federico II, Naples 80131, Italy

## Abstract

Congenital heart disease (CHD) affects eight out of 1,000 live births and is a major social and health-care burden. A common genetic cause of CHD is the 22q11.2 deletion, which is the basis of the homonymous deletion syndrome (22q11.2DS), also known as DiGeorge syndrome. Most of its clinical spectrum is caused by haploinsufficiency of *Tbx1*, a gene encoding a T-box transcription factor. Here we show that Tbx1 positively regulates monomethylation of histone 3 lysine 4 (H3K4me1) through interaction with and recruitment of histone methyltransferases. Treatment of cells with tranylcypromine (TCP), an inhibitor of histone demethylases, rebalances the loss of H3K4me1 and rescues the expression of approximately one-third of the genes dysregulated by *Tbx1* suppression. In Tbx1 mouse mutants, TCP treatment ameliorates substantially the cardiovascular phenotype. These data suggest that epigenetic drugs may represent a potential therapeutic strategy for rescue of gene haploinsufficiency phenotypes, including structural defects.

## Abstract (web summary)

Deficit in transcription factor Tbx1 causes heart defects in humans and mice. Here the authors show that Tbx1 regulates gene expression by recruiting histone methyltransferases that affect chromatin marks, and that a drug inhibiting histone demethylation ameliorates the cardiovascular phenotype in Tbx1 haploinsufficient or hypomorphic mice.

## Graphical abstract

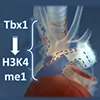

DiGeorge syndrome is the subject of clinical as well as biological interest because of the relatively high frequency of 22q11.2 deletion in humans, which is the main genetic cause, and because it represents the most striking example of a developmental field defect of the cardio-pharyngeal apparatus. The genetic cause of this syndrome has been narrowed down to haploinsufficiency of a single gene, *Tbx1* (refs 1, 2, 3, 4). *Tbx1* mutant mice recapitulate well the cardio-pharyngeal phenotype, thus providing an extraordinary opportunity to dissect genetic and molecular mechanisms coordinating the concerted development of a vital multiorgan system. Developmental defects of this system give rise to the most common congenital abnormalities in humans.

Interestingly, recent studies support the existence of an evolutionary conserved mesodermal cell lineage, named the cardiopharyngeal cell lineage that contributes to the second heart field (SHF) family of multipotent heart progenitors and to branchiomeric muscle^5^. Tbx1 is a major player in the mammalian SHF^6^,^7^,^8^ and in the cardiopharyngeal lineage of *Ciona intestinalis*^9^, but the role that it plays in the mammalian cardiopharyngeal lineage is unclear. Cre/*lox*-based cell fate mapping experiments have shown that descendants of *Tbx1*-expressing cells contribute to the heart, vasculature endothelium and branchiomeric muscle, suggesting that the gene is expressed in this lineage^10^,^11^,^12^.

*Tbx1* encodes a T-box transcription factor and its loss in the SHF leads to reduced cell proliferation, premature differentiation^6^ and reduced contribution of cardiac progenitors to the heart^13^. These abnormalities lead to hypoplasia and lack of septation of the cardiac outflow tract (OFT), inter-ventricular septation defects as well as other morphological abnormalities. Severe abnormalities also occur in other organs derived from the pharyngeal apparatus, for example branchiomeric muscle, pharyngeal arch arteries, thymus, parathyroids and thyroid^1^,^14^,^15^,^16^. Despite its developmental importance, the molecular mechanisms by which Tbx1 operates are unclear.

In this study, we found that Tbx1 binds to regions rich in H3K4me1 and poor in H3K27Ac. Tbx1 regulates positively H3K4me1 enrichment through interaction with histone methyltransferases. Furthermore, inhibition of histone demethylation using a drug led to partial correction of gene dysregulation caused by loss of Tbx1, and to partial phenotypic rescue in *Tbx1* mouse mutants. We conclude that regulation of H3K4me1 enrichment is an important component of Tbx1 function.

## Results

### Tbx1 binds to H3K4me1-rich and H3K27Ac-poor regions

**Figure 1:**
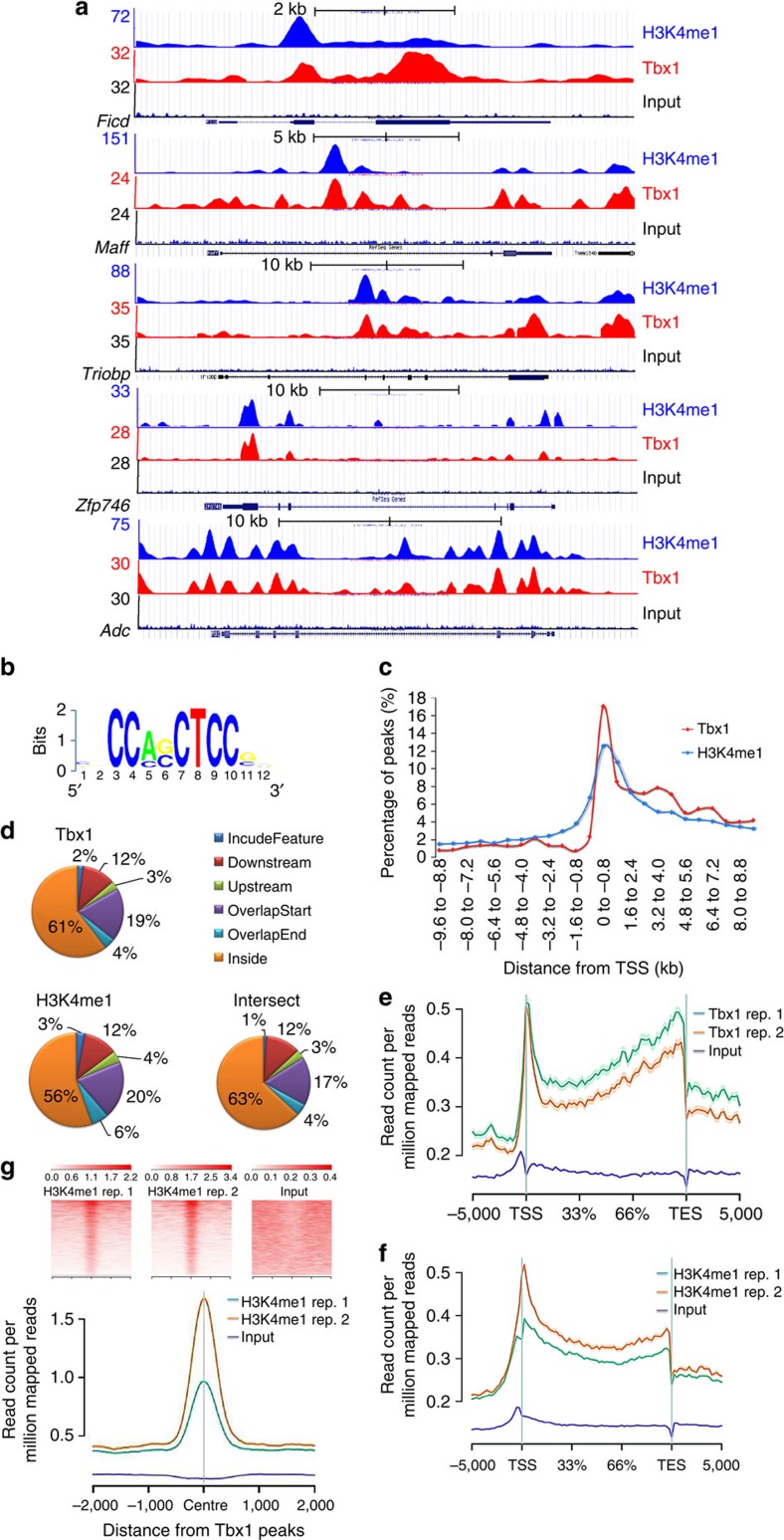
Tbx1-enriched regions co-localize with H3K4me1 enrichment. (**a**) Examples of ChIP-seq data profiles as shown using the UCSC genome browser. Note the similarities between Tbx1 and H3K4me1 signals. (**b**) Sequence logo representing the enriched Tbx1 motif identified by *de novo* motif discovery. (**c**) Distribution of Tbx1 and H3K4me1-binding sites relative to the closest TSS. Note the sharp clustering of Tbx1 peaks within the first 800 bp downstream of the TSS. H3K4me1 peaks have a similar but broader distribution. (**d**) Genomic distribution of Tbx1, H3K4me1 and overlapping Tbx1/H3k4me1 (intersect) binding sites relative to genes. In all cases, the majority of sites is intragenic. Feature id: ensembl gene ID. (**e**,**f**) Map of Tbx1 (**e**) and H3K4me1 (**f**) enrichments relative to the body of RefSeq genes and to 5 kbp upstream of the TSS and 5 kbp downstream of the TES. The transcribed regions were normalized to the same length. The graph shows ChIP-seq data from two independent experiments. Note the highest enrichment downstream to the start site and a gradual increase towards the 3′-end of genes. Shaded areas around each curve indicate the standard errors. (**g**) H3K4me1 occupancy relative to Tbx1 summits in two biological replicates. (top) Heat-map of H3K4me1 ChIP-seq enrichment across Tbx1-binding sites. Each row represents a 4 kb window centred on summits of Tbx1 peaks and extending 2 kb upstream and 2 kb downstream. (bottom) Averaged ChIP-seq enrichment profile across a 4 kb window centred on Tbx1 sites. Thus, H3K4me1 enrichment tends to center around a Tbx1 peak. TES, transcription end sites.

We built an integrated map of Tbx1-binding sites, gene expression, H3K4me1, and acetylation of histone 3 lysine 27 (H3K27Ac) enrichment in differentiating mouse P19Cl6 cells^17^ using chromatin immunoprecipitation (ChIP)-seq and PolyA+ RNA-seq (Supplementary Fig. 1a–c and Supplementary Data 1). Principal component analysis of the transcriptional profiles of our cells and of differentiating mouse embryonic stem cells (ESC)^18^ indicated that P19Cl6 cells used here at the differentiation stage with the highest *Tbx1* gene expression, have a transcriptional profile intermediate between ESC and mesodermal differentiation states (Supplementary Fig. 1d). High-stringency data analysis (Supplementary Data 2, examples in Fig. 1a) identified 2,388 Tbx1-enriched regions (peaks) genome-wide, 50% of which co-localized with regulatory sequences^19^ (*n*=1,149, *P*<0.001, empirical bootsrap approach). We derived a Tbx1 consensus binding motif (Fig. 1b), which was present in 47% of the peaks. We identified PAX4, TFAP2, CREB1, AHR and ATF6 as the most significantly enriched sites adjacent to Tbx1 peak summits^20^ (local enrichment *P* value from 0.004 to 0.015). The 2,388 peaks were associated with 2,390 protein coding genes (Supplementary Data 2), 84% of which were expressed (*P*<10^−10^, Fisher). 71% of Tbx1 peaks were located within H3K4me1-enriched regions (examples in Fig. 1a,g). The likelihood that this co-localization occurs by chance is negligible (*P*<10^−10^, empirical bootstrap approach). Peaks were predominantly intragenic, clustered immediately downstream to the transcription start site (TSS) (Fig. 1c-f). Tbx1 peaks were not enriched for H3K27Ac (Supplementary Fig. 2a,b) indicating that, generally, Tbx1 does not bind active enhancers (defined as H3k4me1+;H3K27Ac+ regions). Indeed, genes associated with Tbx1+;H3K4me1+ regions were expressed at a significantly lower level compared with all the expressed genes or to genes associated with H3K27Ac+;H3K4me1+ regions (*P*<10^−15^, one-side Wilcoxon rank sum test, Supplementary Fig. 2c).

### Tbx1 regulates region-specific H3K4me1 enrichment

**Figure 2:**
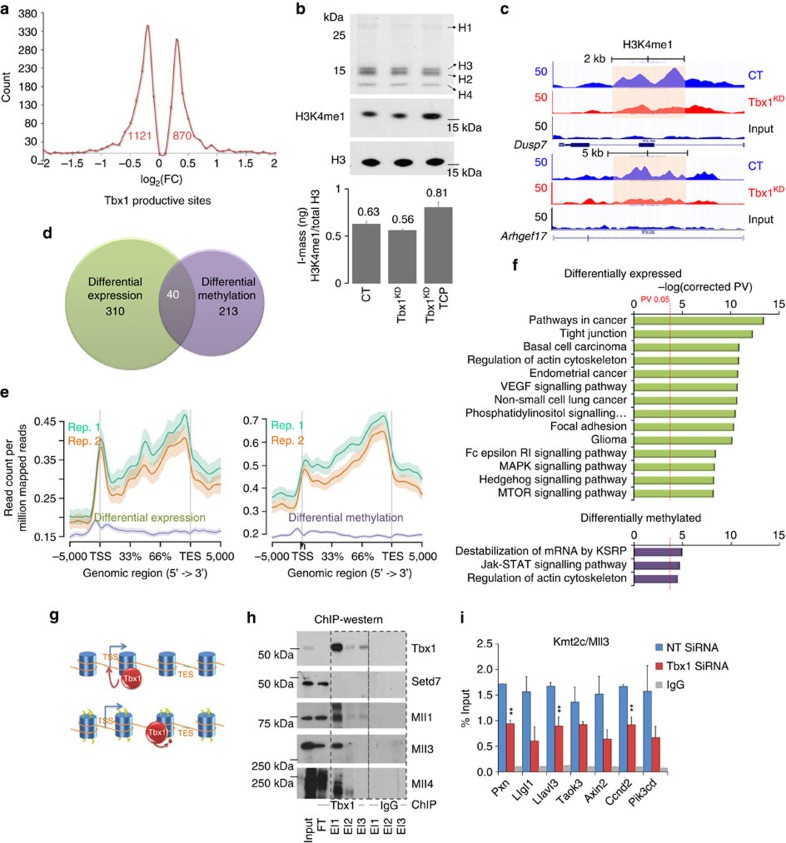
Tbx1 dosage affects gene expression and H3K4 monomethylation. (**a**) Statistically significant gene expression changes in *Tbx1* K/D versus control samples. The graph shows the distribution of the number of genes versus log ratios/FC. (**b**) P19Cl6 cells were transfected with *Tbx1* siRNAs (non-targeting siRNA as control) with and without TCP treatment. (top) Acid-extracted histones from P19Cl6 cells run on a 15% SDS–PAGE and stained with Coomassie Blue dye. (bottom) Isolated histones were subjected to SDS–PAGE and monomethylation at histone H3K4 or total H3 as control were detected with specific antibodies. (**c**) Examples of H3K4me1 ChIP-seq signal distribution in two loci before and after *Tbx1* K/D. The shaded area indicate regions with reduced methylation. (**d**) Venn diagram grouping Tbx1 peaks associated with differentially expressed genes (green) and Tbx1 peaks down-methylated (purple) after *Tbx1* K/D. The two groups are clearly distinct with only a small overlap. (**e**) The graph shows the location relative to the TSS of productive Tbx1 peaks. The peaks associated with differentially expressed genes are clearly closer to the TSS. (**f**) Canonical pathways by InnateDB analysis of differentially expressed (green) and differentially methylated (purple) genes associated to Tbx1 productive sites. (**g**) The cartoon illustrates the two types of response to Tbx1 dosage (DE and differential methylation). (**h**) ChIP–western blot analyses of co-immunoprecipitation experiments with endogenous proteins from P19Cl6 differentiating cells. Immunoprecipitation was carried out using an anti-Tbx1 antibody or rabbit IgG (control). Interacting proteins were eluted from the beads using three consecutive elutions (EL1, EL2 and EL3). Western blots were carried out using anti-Tbx1 (control), anti-Setd7, anti-Kmt2a/Mll1, anti-Kmt2c/Mll3 and anti-Kmt2b/Mll4 antibodies. (**i**) q-ChIP assay on a subset of Tbx1-binding sites in P19Cl6 cells (with and without Tbx1 K/D) using an anti-Kmt2c antibody, followed by qRT-PCR. Error bars: s.e.m.; *n*=3; two-tailed Student's *t*-test. ** *P*<0.01. FC, fold change; q-ChIP, quantitative ChIP.

*Tbx1* knockdown (Supplementary Fig. 1e,f) revealed differential expression (DE) of 1,991 protein coding genes (Fig. 2a and Supplementary Data 3), 13.4% of which (*n*=266) were associated with 350 Tbx1 peaks (Supplementary Data 3). *Tbx1* knock down caused a small but significant overall reduction of H3K4me1 (Fig. 2b), and out of 44,208 H3K4me1-enriched regions, 3,198 had significantly reduced H3K4me1 levels (differentially methylated, DM, regions) (examples in Fig. 2c). Only 246 regions showed increased H3K4me1, indicating that Tbx1 primarily enhances H3K4me1 levels (Supplementary Data 4). A significant proportion of the DE genes were associated with a DM region (20.5%, *n*=408, *P*<10^−3^, hypergeometric test). In addition, 26% of the genes associated with a Tbx1 peak were associated with a DM region. Furthermore, 253 Tbx1 peaks overlapped with a DM region (10.6% of the TBX1 peaks, *P*< 10^−4^, empirical bootstrap approach, Supplementary Data 4). In a time-course experiment, H3K4me1 variation preceded gene expression variation in most cases (Supplementary Fig. 3). In summary, Tbx1 occupies 563 ‘productive' sites (∼24% of the peaks), defined as sites that respond to Tbx1 dosage by altering gene expression (*n*=310), or methylation (*n*=213) or both (*n*=40) (Fig. 2d and Supplementary Data 2). While peaks associated with DE have a similar distribution to that of Tbx1 peaks in general (compare Fig. 2e, left, with Fig. 1e), DM peaks are closer to the transcription end site (Fig. 2e, left). Pathway-enrichment analyses of associated genes produced different results for the two groups of genes (Fig. 2f–g).

### Tbx1 interacts with and recruits histone methyltransferases

We asked how Tbx1 regulates H3K4me1 enrichment. Loss of Tbx1 in P19Cl6 cells or in mouse embryos did not affect H3K4 methyltransferase activity (Supplementary Fig. 4a,b). In addition, DE of genes encoding H3K4 methyltransferases or demethylases does not explain H3K4me1 changes (Supplementary Data 5). In contrast, ChIP–western blotting revealed that Tbx1 colocalizes with at least three H3K4 methyltransferases: Kmt2a, Kmt2b and Kmt2c (Fig. 2h). The expression of the genes encoding these proteins did not change in wild-type (WT) and *Tbx1*^+/−^ embryos (Supplementary Fig. 4c). We pursued Kmt2c/Mll3 and found that Tbx1 and Kmt2c co-immunoprecipitate (Supplementary Fig. 4d). Consistently, quantitative ChIP assays (q-ChIP) revealed that *Tbx1* knockdown lowers Kmt2c levels in individual sites (Fig. 2i). In addition, we found that the transcriptional response to over expression of *Tbx1* of *Axin2* and *Wnt5a* is impaired by *Kmt2c* gene knockdown (Supplementary Fig. 4e). In summary, our data support a mechanism by which Tbx1 regulates H3K4me1 enrichment by recruiting one or more methyltransferases onto chromatin.

### Inhibition of demethylases partially counteracts Tbx1 loss

**Figure 3:**
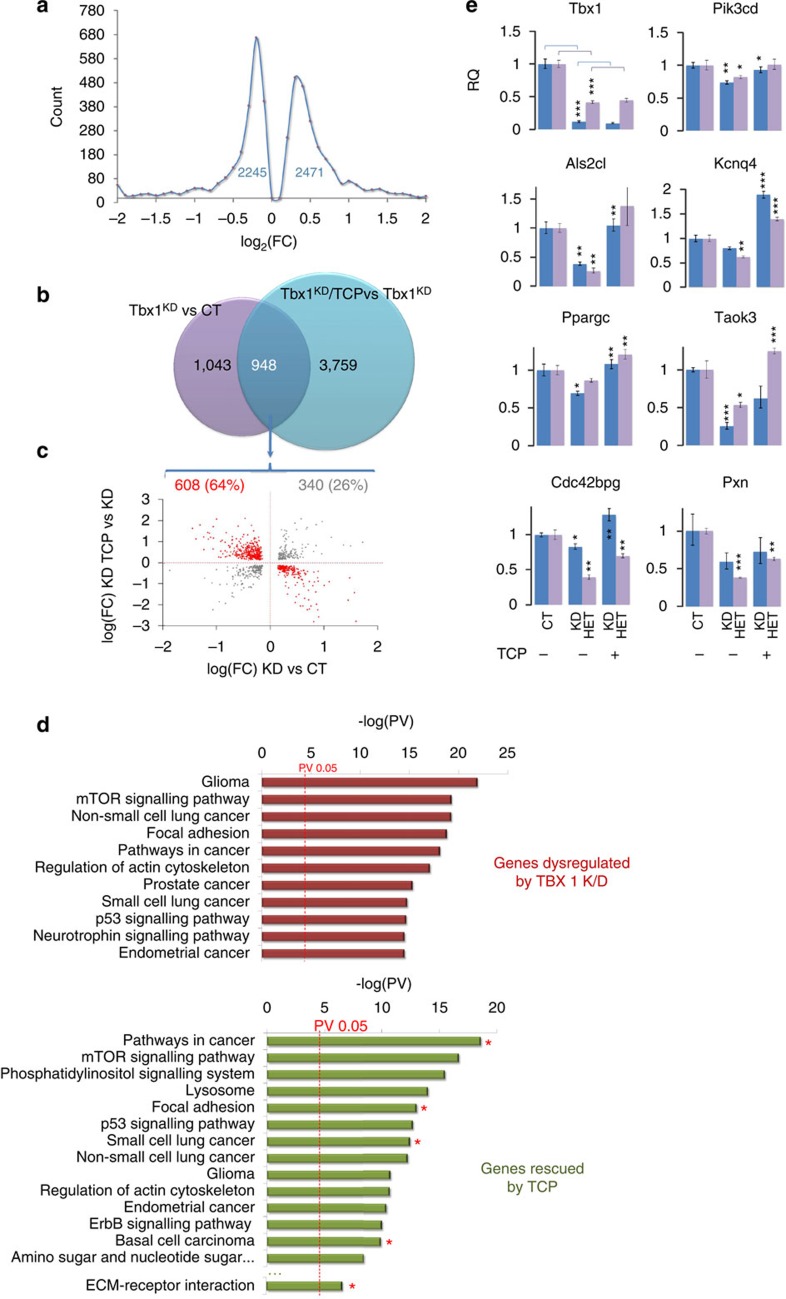
TCP partially rescues gene dysregulation caused by Tbx1 K/D in cell culture. (**a**) Statistically significant gene expression changes in *Tbx1* K/D+TCP versus Tbx1 K/D samples. The graph shows the distribution of the number of genes versus log ratios/FC. (**b**) Venn diagram grouping genes significantly up- or downregulated in *Tbx1* K/D versus control samples (blue) and *Tbx1* K/D+TCP versus *Tbx1* K/D samples. Note the large overlap of 948 genes. (**c**) Scatter plot depicting the DE of 608 genes ‘rescued' by TCP treatment (discordant) and of 246 genes ‘worsened' by TCP treatment (concordant). (**d**) Canonical pathways defined in the InnateDB with significant enrichment of genes differentially expressed after *Tbx1* KD (red, top) and rescued by TCP treatment (green, bottom). Co-occurrences of rescued pathways with *in vivo* TCP treatment (Fig. 4n) are indicated with red asterisks. (**e**) qRT-PCR validation of DE of a subset of rescued genes in P19Cl6 cells (with and without K/D), and WT and *Tbx1*^+/LacZ^ (HET) E9.5 mice embryos, with and without TCP treatment. Asterisks above bars indicated significant differences compared with the control (*P<0.05; **P<0.01; ***P<0.001). Error bars: s.e.m.; *n*=3; two-tailed Student's *t*-test. FC, fold change.

We asked whether drug-induced enhancement of H3K4me1 could partially compensate for reduced *Tbx1* dosage. To test this hypothesis, we used tranylcypromine (TCP), an inhibitor of Lsd1/2 histone demethylases^21^. TCP treatment of differentiating, *Tbx1* knockdown P19Cl6 cells increased the overall H3K4me1 level by 45% (Fig. 2b), and affected the expression of 4,707 protein-coding genes (Supplementary Data 6 and Fig. 3a). Out of 1,991 genes affected by *Tbx1* knockdown, 948 genes (48%) responded to TCP treatment (*P*<10^−10^, hypergeometric test). Strikingly, the expression of 608 of those genes (64.1%) was rescued by TCP treatment, that is, those downregulated by *Tbx1* knockdown were upregulated after TCP treatment, and vice versa (Fig. 3b,c and Supplementary Data 7). Gene pathway analyses of DE genes and TCP-rescued genes revealed that top scoring pathways perturbed by loss of Tbx1 are rescued by TCP treatment (Fig. 3d). We confirmed a subset of rescued genes in P19Cl6 cells and in embryos by quantitative real-time PCR (qRT-PCR) (Fig. 3e). In addition, we found that 30.3% (*n*=184) of the rescued genes was differentially methylated after TCP treatment (Supplementary Data 8), and 45% (*n*=284) of the genes associated with Tbx1 productive sites was differentially methylated by TCP (*P*<10^−10^, hypergeometric test). TCP treatment also caused local differential methylation of 124 Tbx1 productive sites, which, remarkably, were almost all (83%, *n*=103) differentially methylated by Tbx1-dosage modification. Knockdown of Lsd1 and/or Lsd2 recapitulated the effects of TCP on 11 out of 12 genes individually tested (Supplementary Table 1). TCP did not affect the recruitment of Kmt2c to target loci (Supplementary Fig. 4f). In addition, ChIP–western blotting data indicated that Lsd1 can be found in Tbx1-immunoprecipitated chromatin (Supplementary Fig. 4g), explaining why the inhibition of Lsd1 by TCP can have local effects in at least some Tbx1-bound chromatin regions. In summary, TCP, through Lsd1/2 inhibition, rescues a substantial fraction of the transcriptional and H3K4me1 dysregulation caused by *Tbx1* knockdown.

### Demethylase inhibition ameliorates the *Tbx1* mutant phenotype

**Figure 4:**
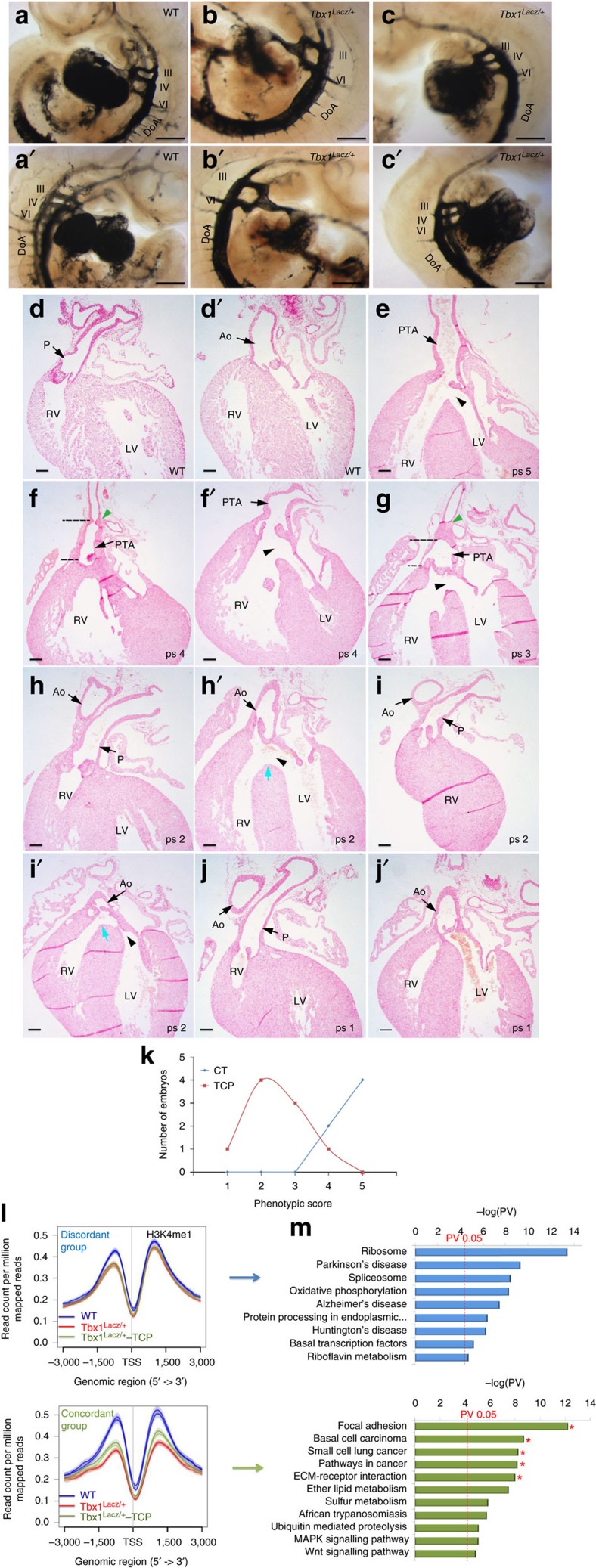
TCP partially rescues the cardiovascular phenotype of Tbx1 mutants. (**a**–**c**′) Ink injection of E10.5 embryos. (**a**,**a′**) IIIrd, IVth and VIth PAAs in WT embryos. (**b**,**b**′) untreated *Tbx1*^*Lacz/+*^ embryo with aplasia of the IVth PAA. (**c**–**c**′) normal IVth PAA in a treated *Tbx1*^*Lacz*/+^ embryo. DoA, dorsal aorta. Scale bars: 100 μm (**a**–**c**'). (**d**–**j**′) Coronal sections of WT (**d**,**d**′), untreated (**e**,**f**) and treated (**g**–**j**) *Tbx1*^*Neo2*/*LacZ*^ hearts (E18.5). (**e**) PTA and VSD (arrowhead) in a *Tbx1*^*Neo2*/*LacZ*^ untreated embryo (ps 5). (**f**,**f**′) untreated *Tbx1*^*Neo2*/LacZ^ embryo with incomplete PTA and VSD (black arrowed); dashed lines: unseptated truncal region; green arrowhead: bifurcation of aorta and pulmonary trunk (ps 4). (**g**) incomplete PTA in a treated *Tbx1*^*Neo2*/*LacZ*^ embryo. The unseptated region (dashed lines) is restricted to the OFT valve region (ps 3). (**h**,**h**′) A treated *Tbx1*^*Neo2*/*LacZ*^ embryo with DORV and VSD (black arrowhead), the tip of the VS is under the left wall of the aorta (blue arrow) (ps 2). (**i**,**i**′) DORV in a treated *Tbx1*^*Neo2*/*LacZ*^ embryo. The tip of the VS is under the middle of the aortic valve (blue arrow), suggesting improved alignment of aorta and LV, compared with the sample in **h**,**h′** (ps 2). (**j**,**j′**) Normal conotruncus in a treated *Tbx1*^*Neo2*/*LacZ*^ embryo (ps 1). Ao, aorta; P, pulmonary trunk. Scale bars: 100 μm (d–j′). Whole mount of samples sectioned in **e**–**j** are shown in Supplementary Fig. 5, **a**–**f**, respectively. (**k**) Phenotypic scores (ps) for treated and untreated *Tbx1*^*Neo2*/*LacZ*^ embryos. (**l**) Genomic distribution of H3K4me1 in two biological replicates of WT (blue), *Tbx1*^*LacZ*/+^ untreated (red) or TCP-treated *Tbx1*^*LacZ*/+^ E9.75 (25 somites) embryos (green) in genes characterized by negative correlation (discordant) or positive correlation (concordant) between expression and methylation in *Tbx1*^*LacZ/+*^ versus WT comparisons. (**m**) Canonical pathways by InnateDB analysis of discordant (blue) and concordant (green) genes. Some of these pathways (asterisks) were also identified in cell culture experiments (Fig. 3d). PV: hypergeometric test, BH correction. DORV, double-outlet right ventricle; LV, left ventricle; RV, right ventricle; VS, ventricular septum; VSD, ventricular septal defect.

We asked whether TCP modifies the *Tbx1* mutant phenotype *in vivo*. We used mouse mutants that express reduced dosages of *Tbx1* mRNA, that is, *Tbx1*^+/*LacZ*^ (50% of WT mRNA level) and *Tbx1*^*Neo2*/*LacZ*^ (15%) (ref. 22), in order to better mimic the human disease, which is caused by gene haploinsufficiency. The most penetrant *Tbx1* haploinsufficiency phenotype is aplasia or hypoplasia of the fourth pharyngeal arch arteries (PAAs), visualized in E10.5 embryos by ink injection^2^. This phenotypic anomaly is due to a requirement for *Tbx1* between E7.5 and E9.5 (ref. 12). We injected TCP into pregnant mice at E7.5, E8.5 and E9.5 and harvested embryos at E10.5. At this stage, the third, fourth and sixth PAAs are clearly visible (Fig. 4a,a′), and only the fourth is affected by *Tbx1* haploinsufficiency. We found that TCP reduces the incidence of fourth PAA abnormalities by ∼50% (*P*<0.001, χ^2^-test) compared with carrier-treated embryos (Fig. 4b,c′ and summary in Supplementary Table 2a). Next, we tested the hypomorphic mutant *Tbx1*^*Neo2/LacZ*^, which presents with a broader range of phenotypes, including cardiac OFT defects^22^. We crossed *Tbx1*^*Neo2*/+^ with *Tbx1*^+/*LacZ*^ mice and injected pregnant females with TCP or carrier at E7.5, E8.5, E9.5 and E10.5 (the requirement for *Tbx1* in OFT development is more prolonged than the one for fourth PAA development^12^). We examined a total of 15 *Tbx1*^*Neo2/LacZ*^ E18.5 embryos, six controls and nine treated with TCP (results summary in Supplementary Table 2b). We scored OFT phenotypic severity from 1 to 5, where 1 is morphologically normal and 5 is the most severely affected, in *Tbx1*^*Neo2/LacZ*^ embryos. Four out of the six control embryos had persistent truncus arteriosus (PTA, score 5, Supplementary Fig. 6a). Two others had a milder form of PTA consisting of a failure of conal septation (thus unseptated OFT valve) but only partial failure of truncal septation (Supplementary Fig. 6b, score 4), consistently with reported data^22^. The average score for the control group was 4.7. In the treated group, we did not observe any score 5 sample, only one case with score 4, while three embryos had a milder form of PTA consisting of a failed conal septation but complete truncal septation (resulting in complete separation of the aorta and pulmonary trunk that merge in a single OFT valve) (Supplementary Fig. 6c, score 3). Four of the TCP-treated embryos had rescued conal and truncal septation, but abnormal connection of the OFT with the ventricles, resulting in a double-outlet right ventricle (Supplementary Fig. 6d,e, score 2). Finally, one treated embryo had a morphologically normal heart (Supplementary Fig. 6f, score 1). The average score for the treated group was 2.4. Histology confirmed the above diagnoses and determined that all of the *Tbx1*^*Neo2*/*LacZ*^ embryos (treated and untreated) presented with ventricular septal defects, with the exception of the treated heart already diagnosed as normal, which had normal septa (Fig. 4d–j′). Treated and untreated embryos were not distinguishable by external appearance, and none had thymus (Supplementary Fig. 7a–c). All treated and untreated WT and heterozygous embryos had morphologically normal external appearance and OFT phenotype. A group of WT TCP-treated females was allowed to deliver, and they gave birth to normal pups, which grew normally and were fertile (*n*=16), suggesting that the TCP treatment alone does not affect development.

Thus, under the conditions tested, TCP treatment ameliorated substantially the fourth PAA and OFT phenotypes of *Tbx1* mutants (Fig. 4k and Supplementary Table 2). The effect of TCP on H3K4me1 dosage was detectable on embryo tissue (Supplementary Fig. 5). We performed qRT-PCR of treated and untreated *Tbx1*^+/*LacZ*^ and *Tbx1*^*Neo2/LacZ*^ E9.5 embryos but could not detect any significant difference in *Tbx1* expression (Fig. 3e top left panel and Supplementary Fig. 8), indicating that the rescue is not due to increased expression of the *Tbx1* gene.

### Genes responsive to *Tbx1* haploinsufficiency and to TCP

We next performed H3K4me1 ChIP-seq and RNA-seq on whole E9.75 (25 somites) WT and *Tbx1*^*+/LacZ*^ embryos, injected with carrier or TCP at E7.5, E8.5 and E9.5. We integrated ChIP-seq and RNA-seq data^23^,^24^ (expressed genes listed in Supplementary Data 9) and identified 855 DE and DM genes (Supplementary Data 10; differentially expressed genes listed in Supplementary Data 11). This group of genes comprises two subgroups, one (*n*=336, Supplementary Data 12) in which expression and methylation moved in the same direction (both up or both down, ‘concordant' group), and one (*n*=519) in which the two parameters moved in opposite directions (‘discordant' group). Although by definition, both groups responded to Tbx1 dosage, there were important differences, illustrated in Fig. 4l. The discordant group (top) showed only modest H3K4me1 variation that was restricted to sequences upstream to the TSS. In contrast, the concordant group (Fig. 4l, bottom) showed a strong response to *Tbx1* dosage, both upstream and downstream to the TSS. Even more striking was the differential response of the two groups to TCP treatment in *Tbx1*^*+/LacZ*^ embryos (green lines in Fig. 4l). Indeed, the discordant group showed no detectable response, while the concordant genes responded to TCP by increased methylation to a level intermediate between the WT and untreated heterozygous mutants. Thus, these experiments identified a group of 336 genes, the expression and H3K4me1 of which are perturbed by *Tbx1* heterozygosity, and that are partially rescued by TCP treatment. One-fourth of these genes (*n*=84) were associated with Tbx1 peaks in P19Cl6 cells (Supplementary Data 12). Remarkably, gene pathway analysis of genes rescued by TCP in the P19Cl6 cell culture model and in embryos provided overlapping results (asterisks in Fig. 4m, bottom, compare with Fig. 3d, bottom), suggesting that TCP rescues specific pathways perturbed by reduced dosage of Tbx1 in both systems. Next, we tested treated and untreated *Tbx1*^*Neo2/LacZ*^ E9.5 embryos and found that the expression of *Fgf8* (a gene known to be downregulated in *Tbx1* mutants^25^) was upregulated after TCP treatment (Supplementary Fig. 9a,a′). In addition, cell proliferation in the SHF (typically reduced in *Tbx1* mutants^6^,^26^) was increased in treated *Tbx1*^*Neo2/LacZ*^ embryos (Supplementary Fig. 9b–d). Furthermore, cell shape changes identified in the SHF of *Tbx1* mutants^27^ were ameliorated after TCP treatment of *Tbx1*^*Neo2/LacZ*^ embryos, as determined by aPKCζ immunofluorescence (Supplementary Fig. 9e–g).

## Discussion

We show that the localization of the Tbx1 protein on chromatin is predominantly within genes that are transcribed at a low level. This is consistent with the lack of H3K27Ac enrichment, a mark of transcriptional activation, at Tbx1-occupied sites. Furthermore, *Tbx1* knockdown in cells, and *Tbx1* heterozygosity in embryos, cause relatively small changes in gene expression that can be in either direction in a similar measure. Thus, *Tbx1* is neither a strong transcriptional activator nor a strong transcriptional repressor. In contrast, the effect of Tbx1 dosage on H3K4me1 is clear: it is a positive regulation. In addition, our time-course experiments reveal that after *Tbx1* knockdown, reduced monomethylation precedes gene expression variation at most of the tested loci; sometimes the two events were detected at the same time point, but in no case did we see transcriptional variation preceding methylation change. Thus, Tbx1 has an early effect on histone modification, while the transcriptional changes are probably downstream consequences co-driven by other transcription factors or co-factors. Given this scenario, we propose that Tbx1 is a priming factor that keeps targeted chromatin ‘available' to other regulatory factors, which may be activators or repressors. This is an attractive hypothesis that is consistent with a role in cell lineage determination or maintenance. Indeed, in the mouse, Tbx1 is critical for cell fate determination in the inner ear^28^, while in *Ciona*, the Tbx1 homologue, known as Tbx1/10 determines pharyngeal muscle fate^9^.

How does Tbx1 regulate H3K4me1 enrichment? We found no evidence that this effect is mediated by the regulation of genes encoding histone methylation or demethylation enzymes, nor did we detect an effect on methyltransferase enzymatic activity. Instead, we found that the chromatin context within which Tbx1 operates is enriched in histone methyltransferases. Furthermore, Tbx1 depletion reduces histone methyltransferase enrichment at selected loci, suggesting recruitment activity, and explaining local H3K4me1 depletion in the absence of Tbx1. The fact that the histone demethylase Lsd1 is also found in Tbx1-enriched chromatin suggests that Tbx1 may be at the fulcrum of the balance between methylation and demethylation of its target chromatin. This leads us to propose a mechanism by which *Tbx1* haploinsufficiency is the result of an imbalance between the two activities. However, we acknowledge that this mechanism probably does not account for all the molecular functions of Tbx1 in the chromatin context, and thus further studies will be necessary to elucidate fully its role.

The proposed mechanisms described above led us to test whether inhibiting the enzymatic activity of Lsd1/Lsd2 can compensate for reduced H3K4me1 enrichment caused by lowered Tbx1 dosage. The rationale being that inhibition would re-establish the balance between methylation and demethylation. Experimental results are supportive of this rationale because rescue was highly significant, albeit incomplete. Interestingly, the rescue occurred at different levels, including increased H3K4me1, partial correction of transcriptional dysregulation, improvement of SHF cell proliferation and cell morphology and amelioration of structural heart defects. This supports the view that the regulation of H3K4me1 enrichment is central to the biological role of Tbx1.

TCP-dependent phenotypic rescue may be incomplete for several reasons. Firstly, Lsd1/Lsd2 may not be the only demethylase that targets Tbx1-responsive regulatory elements. Secondly, Tbx1 may regulate transcription through additional mechanisms and not only by H3K4me1 enrichment. Thirdly, Tbx1 can regulate transcription indirectly by binding other transcription factors without binding to DNA, as previously shown^6^,^29^.

**Figure 5:**
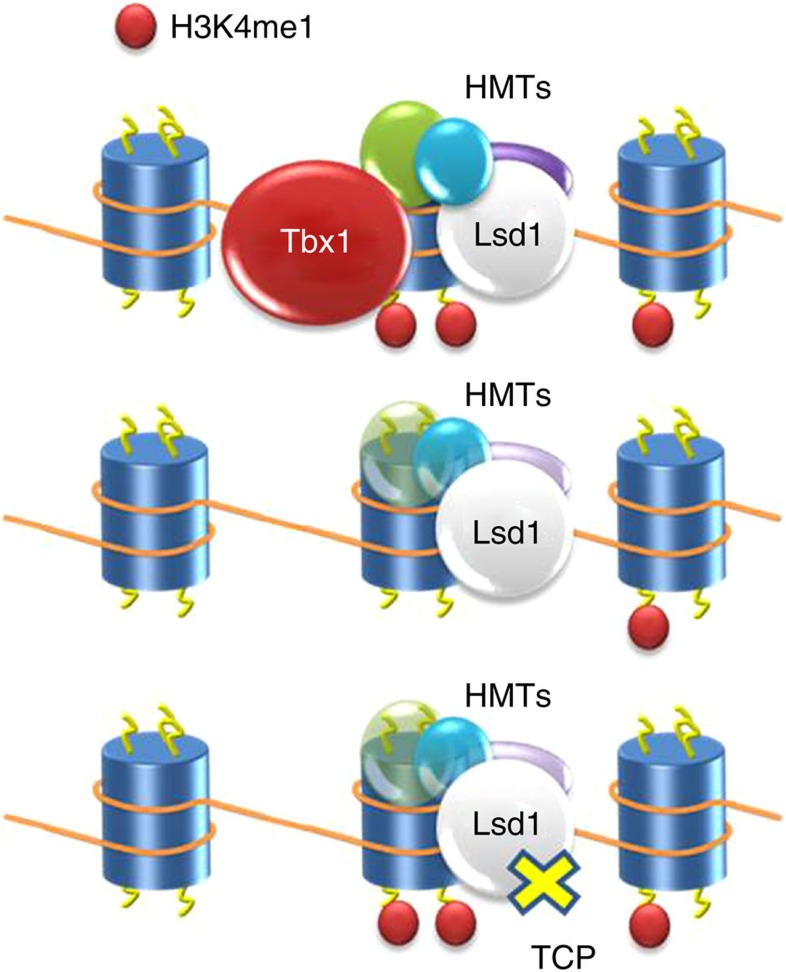
Cartoon showing a working model for Tbx1 interactions with chromatin and TCP rebalancing effect. In the WT state (top row), Tbx1 promotes H3K4me1 deposition (small red dots) through interaction with histone methyltransferases (HMTs). When Tbx1 is absent or of lowered dosage (middle row), H3K4me1 enrichment is lowered because of reduced recruitment of HMTs. TCP treatment (bottom row) re-establishes H3K4me1 by inhibiting the activity of the Lsd1 demethylase. Blue cylinders indicate histone octamers.

Figure 5 shows a working model for the interactions between Tbx1 and chromatin.

Analysis of pathways perturbed by the mutation of a given gene provides useful insights into pathogenetic mechanisms. However, a simple WT vs mutant comparison cannot tell us which of the pathways is critical for the mutant phenotype. Rescue experiments may provide additional precious information to this effect. We performed pathway analyses in cellular and embryonic models using TCP rescue and, remarkably, we found substantial overlap among the rescued pathways identified in the two models, suggesting robustness of the approach. Some of the pathways identified were unexpected, for example those implicated in cell morphology and adhesion, and cancer. However, recent data indicate loss of polarity in SHF cells of *Tbx1* mutants^27^. The planar cell polarity pathway, although not in the top scoring ones, was confirmed to be regulated by Tbx1 as the genes *Wnt5a* and *Ror2* (encoding ligand and receptor, respectively) were directly targeted. Also of interest is the strong significance for a basal cell carcinoma pathway because Tbx1 has been recently implicated in this type of tumour^30^. Furthermore, rescue in the cell model revealed the implication of p53 pathway; in an independent study, we reported that suppression of p53 leads to partial rescue of the *Tbx1* mutant phenotype^31^, thus providing an *in vivo* validation of the relevance of this pathway. Interestingly, we did not confirm that Tbx1 is directly involved in cell proliferation and differentiation, which are two cellular phenotypes affected in *Tbx1* mutants. While the interaction with the p53 pathway may partly explain these phenotypes, it is also possible that they are an indirect consequence of interactions with other cellular functions such as cytoskeletal rearrangement, cell adhesion and motility.

Our studies were carried out in a cell line and in whole-embryo material and were designed to identify general mechanisms of Tbx1 function and genetic pathways altered by its loss. The identification of cell lineage-specific targets will require further studies using homogenous cell types. Nevertheless, our results have defined a novel model for the molecular function of Tbx1 and identified genetic pathways that open a new window onto the cellular functions of this transcription factor.

Last but not least, our data provide a proof of concept that gene haploinsufficiency is a druggable epigenetic condition that can be targeted to alleviate its phenotypic consequences.

## Methods

### Mouse lines and *in vivo* drug treatment

Mice carrying the alleles *Tbx1*^*lacZ*^ (^2^) and *Tbx1*^*neo2*^ (^26^) were maintained in SPF facilities in a C57Bl6 background and were genotyped by PCR using the primers listed in Supplementary Data 13. For timed crosses, developmental stage was evaluated by considering the morning of vaginal plug as embryonic (E) day 0.5. TCP was dissolved in 0.9% w/v NaCl solution. Intraperitoneal injections of 10 mg kg^−1^ body weight of TCP were administered at E7.5, E8.5 and E9.5 (and E10.5 for experiments of OFT phenotype rescue) to pregnant mice in the early morning. Control animals were injected with an equal volume of carrier (sterile 0.9% NaCl). Mouse work was done according to current regulations under the Animal Protocol 257/2015-PR approved by the Italian Ministry of Health.

### Mouse phenotyping

All phenotyping tests (morphology, immunohistochemistry and immunofluorescence) have been performed blind of genotype information. To visualize the pharyngeal arch arteries, we injected India ink into the heart of E10.5 embryos soon after harvest. Subsequently, embryos were fixed in PBS-buffered 4% formaldehyde, dehydrated and cleared in 1:1 benzyl benzoate:methyl salicylate. Images were taken using Zeiss Stemi2000C microscope and Axiovision AC 4.8 image analysis software were used to perform acquisition. Z-stacks were taken at 10 intervals through the thickness of the entire embryo and three-dimensional reconstructions of Z-stacks were done using CombineZP software.

E18.5 embryonic hearts were embedded with paraffin and coronally sectioned into 10 m sections using a Leica microtome. Sections were counterstained with nuclear fast red.

### Immunohistochemistry and mitotic cell counting

6-μm serial sections from paraffin blocks were immunostained with a rabbit anti phosphorylated Histone H3 (Ser10) antibody (Cell Signaling; 1:200 dilution) and Vectastain elite ABC kit (Rabbit IgG) (Vectorlabs #PK-6101) to detect mitotic cells. Antigen retrieval was carried out in 1 mM EDTA/0.05%Tween (PH 8.0) buffer for 15 min. After blocking in 2% goat serum/PBST (PBS containing 0.05% Tween20), sections were incubated with the primary antibody overnight at 4 °C and then with 3% H_2_O_2_/PBS for 10 min to quench endogenous peroxidase activity. Next, sections were incubated with the secondary antibody in GTVision I Detection System/Mo&Rb (Gene Tech) at room temperature (RT) for 30 min and immunoreaction was visualized using a DAB kit (Shanghai Weiao Biotech). Sections were dehydrated, mounted and scored under a Leica DM6000B microscope. We immunostained three pairs of somite-matched E9.5 (24 somites) embryos cut in sagittal sections. Five consecutive sections centred on the SHF were scored for each embryo. Positive cells in the SHF (mesodermal cells under OFT) were counted; the bottom boundary of the scored area was arbitrarily set 100 μm caudal to the lower border of the OFT.

### Immunofluorescence

E9.5 embryos (24 somites) were paraffin-embedded and sectioned. Antigen retrieval was carried out in 1 mM EDTA/0.05%Tween (PH 8.0) buffer for 15 min and immunostaining was performed using an antibody anti aPKCζ (Santa Cruz sc-216, 1:50) and Alexa fluor 594 Goat anti-rabbit IgG (H+L) (Life Technologies #A11012 1:400) as secondary antibody Images were collected using a confocal microscope. The cytoplasmic membrane signal length around SHF cells was evaluated by segmenting the antibody signal and measuring its length on 180 cells from 3 embryos for each experimental point (WT, PBS-treated *Tbx1*^*Neo2/LacZ*^, and TCP-treated *Tbx1*^*Neo2/LacZ*^). Results were evaluated using the Student's t test.

### P19Cl6 cell differentiation

P19CL6 cells were obtained from RIKEN BioResource Center (Ibaraki, Japan, # RCB2318). Cells were mycoplasma-free and were grown in Dulbecco-Modified Minimal Essential Medium supplemented with 10% fetal bovine serum. For differentiation and transient transfection, cells were plated at a density of 5.0 × 10^5^ cells per well on a 35-mm tissue culture dish containing 25 pmol of a pool of Silencer Select Pre-Designed Tbx1 SiRNA (Life Technology) and 7.5 μl of Lipofectamine RNAiMAX Reagent (Life Technology) diluted in 500 μl of Opti-MEM Medium. Cells were incubated at 37 °C in 5% CO_2_. Twenty hours later, the medium was replaced with one containing 10 μM 5-Azacytidine and 1 mM TCP or saline solution for 24 h. (ref. 17). After 24 h, the cells were collected and processed for further analysis.

### Histone purification

For histone extraction^32^, P19Cl6 cells were washed with PBS and collected by centrifugation using a refrigerated centrifuge at 300*g* for 10 min. The pellet was incubated for 30 min in hypotonic lysis buffer (10 mM Tris-Cl pH 8.0, 1 mM KCl, 1.5 mM MgCl_2_, 1 mM DTT and protease inhibitors), nuclei were resuspended in acid-extraction buffer (0.4 N H_2_SO_4_), and the extracted histones were precipitated with trichloroacetic acid and resuspended in deionized water for western blot analysis with anti H3K4me1 antibody (Abcam, #ab8895; 1:2,000) and anti-Histone H3 antibody (Abcam, #ab1791; 1:5,000) and Amersham ECL Rabbit IgG, horseradish peroxidase (HRP)-linked whole Ab (GE Heathcare, #NA934V; 1:10,000) as secondary antibody.

Uncropped scans of all the autoradiographies shown in this work are reported in Supplementary Fig. 10.

### Chromatin isolation

Chromatin isolation was performed as described previously^33^. Briefly, P19Cl6 cells were washed with PBS and collected by centrifugation. The pellet was resuspended in buffer A (10 mM HEPES, (pH 7.9), 10 mM KCl, 1.5 mM MgCl_2_, 0.34 M sucrose, 10% glycerol, 0.1% Triton X-100, 1 mM DTT and protease inhibitors). Nuclei were collected in pellet 1 (P1) by low-speed centrifugation. The supernatant (S1) was further clarified by high-speed centrifugation (15 min, 20,000 × g, 4 °C) and nuclei were lysed in buffer B (3 mM EDTA, 0.2 mM EGTA, 1 mM DTT, protease inhibitors). Insoluble chromatin was collected by centrifugation, and the final chromatin pellet (P3) was resuspended in buffer A plus 1 mM CaCl_2_ and 0.2 U of micrococcal nuclease (Sigma). After incubation at 37 °C for 1 min, the nuclease reaction was stopped by the addition of 1 mM EGTA. The immunoblotting detections were performed with the anti Tbx1 antibody (Abcam, #ab18530; 1:1,000), anti Lamin-B (Santa Cruz, #sc-6216; 1:1,000) and anti-Histone H3 antibody (Abcam, #ab1791; 1:5,000). Amersham ECL Rabbit IgG, HRP-linked whole Ab (GE Heathcare, #NA934V; 1:10,000) and HRP-conjugated Anti-Goat IgG secondary antibody (R&D Systems, #HAF109; 1:5,000) as secondary antibodies.

### Histone methyltransferase activity assay

Fresh nuclear extracts were isolated from P19Cl6 cells transfected with non-targeting siRNA or Tbx1 siRNA and from WT, Tbx1^+/−^ and Tbx1^−/−^ E9.5 embryos using NE-PER Nuclear and Cytoplasmic Extraction Reagents (Life technologies). Histone methyltransferase activity was measured using the Histone H3 (K4) Methyltransferase Activity Quantification Assay Kit (Abcam) following manufacturer's protocol.

### Co-immunoprecipitation

For co-immunoprecipitation experiments, 100 μl of protein-G Dynabeads (Life Technology, #10004D) was crosslinked to 20 μg of anti-Tbx1 antibodies (Abcam, # ab18530). Crosslinking was performed as per the manufacturer's instructions. As a negative control, 20 μg of Rabbit Control IgG (Abcam, #ab46540) was added to reaction mixture to control for proteins interacting nonspecifically with the beads. Following crosslinking, nuclear extracts of P19Cl6 cells (D1) were added to the beads and incubated overnight at 4 °C, washed six times with PBS-0.1% Tween-20. Two consecutive elutions were performed with 0.1 M Glycine pH2.5 and immediately neutralized with Tris-HCl, pH 8.0. Samples were subjected to SDS–polyacrylamide gel electrophoresis (SDS–PAGE) and proteins were transferred into polyvinyldene difluoride (PVDF) membrane for western blotting analyses with anti-KMT2C/MLL3 antibodies (Abcam #ab71200 or #ab71200, 1:1,000) and Amersham ECL Rabbit IgG, HRP-linked whole Ab (GE Heathcare, #NA934V; 1:10,000) as secondary antibody.

### RNA extraction and q-RT-PCR

Seven hours post last injection of TCP or saline solution, WT and *Tbx1*^*+/LacZ*^ E9.5 mouse embryos were dissected, frozen in liquid nitrogen and stored at −80 °C. RNA was extracted from P19Cl6 cells and mouse embryos using 1 ml of TRI-Reagent (Ambion/Applied Biosystems). RNA was treated with DNA-free Kit (Ambion/Applied Biosystems) and visualized on a RNA denaturing gel. cDNA was synthesized from 2 μg total RNA using the High Capacity cDNA Reverse Transcription Kit (Applied Biosystems). Target cDNA levels were evaluated by qRT-PCR in 20 μl reactions containing 1 × SYBR green (FastStart Universal SYBR Green Master (Rox), Roche). Results were normalized against *Rpl13a* and compared by relative expression and the delta-delta-cycle threshold method for fold change calculations with the StepOne v2.3 software (Applied Biosystems). The list of primers used in this study is in Supplementary Data 13.

### Chromatin immunoprecipitation

P19Cl6 cells were cross-linked with 1% formaldehyde directly in a dish for 15 min at RT. Cells were then washed two times with PBS, harvested, counted using a haemacytometer chamber and pelleted. ChIP experiments using embryo tissue was carried out as follows. WT and *Tbx1*^*Lacz*/+^ E9.5 mouse embryos were dissected, fixed in 1% formaldehyde at RT for 10 min and then glycine was added to stop the reaction to a final concentration of 0.125 M for 5 min. ChIP was performed with a True MicroChIP kit (Diagenode) according to the manufacturer's protocol.

The cell pellet was suspended in 6 × volumes of cell lysis buffer (10 mM HEPES, 60 mM KCl, 1 mM EDTA, 0.075% v/v NP40, 1 mM DTT and 1 × protease inhibitors, adjusted to pH 7.6) in a 1.5 ml tube incubating on ice for 15 min. The suspension was then homogenized using a tight-fitting Dounce homogenizer to release nuclei and then centrifuged. Isolated nuclei were suspended in 130 μl of Swelling Buffer (50 mM Tris-HCl, pH 8.0, 10 mM EDTA, 0.8% SDS), homogenized again with a Dounce device and incubated for 30 min on ice. The chromatin was then sonicated in microtubes using the Covaris S2 instrument to obtain 100–300 bp fragments (Duty Cycle: 5%, Cycles: 5, Intensity: 3, Temperature (bath): 4 °C, Cycles per Burst: 200, Power mode: Frequency Sweeping, Cycle Time: 60 seconds, Degassing mode: Continuous). After centrifugation at 20,000*g* at 4 °C for 5 min, soluble chromatin (an equivalent amount of ∼25 μg of DNA) was incubated with 10 μg of an anti Tbx1 antibody (Abcam, #ab18530; 10 μg ml^−1^), 3 μg of an anti H3K4me1 antibody (Abcam, #ab8895; 3 μg ml^−1^) or normal rabbit IgG (Santa Cruz Biotechnology, #2027; 10 and 3 μg ml^−1^, respectively) and then incubated with 20 μl of protein A/G PLUS agarose (Santa Cruz Biotechnology) pre-adsorbed with sonicated single stranded herring sperm DNA and BSA. Samples were extensively washed and incubated in an elution buffer (1% SDS and 0.1 M NaHCO_3_) at 30 °C for 20 min. Crosslinking of protein-DNA complexes was reversed at 65 °C ON, followed by treatment with DNase-free RNase A for 30 min at 37 °C and 100 μg ml^−1^ proteinase K for 2 h at 55 °C. DNA was extracted three times with phenol/chloroform and precipitated with ethanol. The IgG negative control was not sequenced but was used in a first step ChIP quality assessment. Quantitative ChIP primers are listed in Supplementary Data 13.

### ChIP–western blot

For western blot analysis of chromatin immunoprecipitation, after washing the immunoprecipitates, 4 × reducing SDS Loading Dye (500 mM Tris HCl, pH 6.8, 8% SDS, 40% glycerine, 20% β-mercaptoethanol, 5 mg ml^−1^ bromophenol blue) was added to the beads as well as to the lysates and the supernatants and samples were incubated at 99 °C for 20 min and loaded onto SDS–PAGE, proteins were then transferred into PVDF membrane for western blotting analyses with anti Tbx1 antibody (Abcam, #ab18530, 1:1,000 or LifeSpan Biosciences LS-C31179/7296, 1:500), anti-SETD7 antibody (ab124708, 1:500), anti MLL1 Antibody (Bethyl Laboratories, A300-074A, 1:1,000), anti-KMT2C/MLL3 antibody (ab71200, 1:500), anti-KMT2B/MLL4 antibody (ab104444; 1:500) and Amersham ECL Rabbit IgG, HRP-linked whole Ab (GE Heathcare, #NA934V; 1:10,000) as secondary antibody.

### Sequencing library preparation

One to ten nanograms of ChIP DNA or input samples were prepared for Solexa and Solid sequencing using Chip-Seq Sample Preparation kit (IP-102-1001) and TruSeq SBS kit v3 HS (FC-401-3002) (50 cycles) or NEB Next DNA Simple Prep Master Mix set 3. Libraries were quantified with Experion DNA 1K Analysis kit (7007107) and Qubit DSDNA HS Assay kit (Q32851). DNA sequencing was carried out using the Illumina/Solexa Genome Analyzer sequencing system or the Solid 4 system.

### ChIP-seq data analysis

We sequenced two biological replicates and three input samples, which were pooled. For each replicate, we have used a range 1.7 × 10^7^–2 × 10^7^ mapped sequence reads. We used an ABI SOLiD platform for single-end reads of length 50 bp, subsequently we cut from the 5′ end to 35 bp after a quality control. We also used an Illumina platform for single-end reads of length 50 bp and two biological replicates for each sample and input.

For read alignment and peak calling, sequence reads were mapped to the mouse genome (mm9) using Bioscope (version 1.3.1) for SOLiD data and Bowtie (version 0.12.9) for Illumina data. We used default parameters allowing up to two mismatches for 35 bp reads and up to three mismatches for 50 bp reads. Then, we kept only uniquely mapped reads. Finally, PCR duplicated reads were filtered out retaining only one mapped read per starting position per strand. To identify enriched regions, we used MACS (version 1.4.2) with Tbx1 ChIP data, and SICER4 (version 1.1) for H3K4me1 data. In both cases, peaks were detected by comparing ChIP samples versus Input. Then, we used Bedtools^34^ to identify sub-regions consistently enriched in biological replicates. We then carried out a filtering procedure. For each potential region of Tbx1 enrichment, we computed an enrichment score as follows: the number of reads in each region was calculated, this value was divided by the total number of non-duplicated mapped reads and by the peak's length, and multiplied by the scale factor of 10^9^ to normalize counts across samples and regions. The resulting value, called enrichment score, was used to filter out the potential peaks. A peak was considered ‘golden' if its enrichment score was greater or equal to 0.7 in both ChIP replicates and at least 4 reads were observed in the Input. In this way, 2,388 enriched regions of Tbx1 were identified as golden peaks and used for all further analyses. Binomial test (one side) was used to assess the significance of non-equal chance association between Tbx1 peaks with or without H3K4me1 enrichment. To account for enriched region sizes in evaluating the overlap we used the bootstrap approach. In particular, we generated random regions of the same size of H3K4me1 enriched regions and with the same number of intervals for each chromosome, and then we computed the number of Tbx1-enriched regions intersecting the randomly generated ones. The process was reiterated 1,000 times to estimate the co-localization *P* value. To assign genes to Tbx1 peaks, we computed the distance (upstream and downstream) between each peak centre and the nearest TSSs using ChIPpeakAnno (version 2.6.1) (ref. 35). Mouse annotation data including TSS (version NCBIM37) was downloaded from the BioMart database (http://www.ensembl.org/Mus_musculus/Info/Index).

Differentially enriched regions between NT SiRNA and Tbx1 SiRNA, Tbx1 SiRNA and Tbx1 SiRNA+TCP conditions were identified using SICER-df. The co-occurrence of Tbx1 binding and decreased H3K4me1 enrichment after Tbx1 knockdown was evaluated measuring the overlap between the differentially enriched regions with the Tbx1 golden peaks (extended up to 300 bp). The bootstrap approach was used to evaluate statistical significance.

The DNA sequences of Tbx1 golden peaks were used to determine *de novo* a consensus motif. We used RSAT^36^ for identifying *de novo* motifs, where the parameter *Markov order (m)* was set to ‘more stringent' and the other parameters were used with default values.

### RNA-Seq data analysis

To compare differentially expressed polyadenylated genes, six RNA samples were used for libraries preparation with the Illumina's strand specific RNA seq protocol, barcoded and pooled in one lane. The raw data for the high-throughput sequencing of cDNA were generated with Illumina platform for strand specific single-end reads of length 50 bp. Two biological replicates were sequenced for each sample. For each replicate, we have used 2.3–3.3 × 10^7^ mapped sequence reads.

Reads were mapped to the mouse genome (mm9) by using TopHat2 (version.2.0.7) (ref. 37), using the annotation as guide. All other parameters were used with their default values. The annotation, Mus_musculus.NCBIM37.67.gtf, was downloaded from http://www.ensembl.org. Gene expression levels were estimated for each sample in term of Fragments Per Kilo base of exon model per Million mapped reads (FPKM) using Cufflinks (version 2.1.1) (ref. 37) and the annotation as guide. In this context, we studied only protein coding genes. Therefore, the annotation file was obtained from the original annotation file retaining only protein-coding genes, the remaining annotated entries were masked. We considered 22,807 protein-coding genes. For each gene, we tested the significance of its expression in all samples with 95% confidence. To this purpose, Cufflinks' confidence intervals of FPKM were used. Genes whose confidence interval contained 0 in both replicates were considered not expressed in that condition. Genes reported as not expressed in all conditions were considered not expressed.

The differentially expressed genes between NT SiRNA and *Tbx1* SiRNA, and between *Tbx1* SiRNA and *Tbx1* SiRNA+TCP conditions were identified using edgeR (version 3.4.0) (ref. 38) using default parameters. Gene count matrix was obtained using HTSeq (version 0.5.4) (http://www-huber.embl.de/users/anders/HTSeq/).

The row counts were normalized using a trimmed mean of M-values between each pair of samples. The experimental variance was estimated before applying the edgeR test, using the Common Dispersion and Tagwise Dispersion methods. Then, the BH algorithm was used to control the false discovery rate^39^. Genes with adjusted *P*-values<0.05 were considered DE.

To study the correlation between Tbx1 occupation and gene expression, we annotated with ChIPpeakAnno the protein coding genes with the Tbx1 *golden* peaks. The association between the expressed genes and the annotated peaks was estimated with Fisher's exact test (two-sided). To evaluate the transcriptional response to TCP treatment after the reduced dosage of Tbx1, we selected genes differentially expressed after Tbx1 knockdown and compared their expression to that of genes differentially expressed after TCP treatment. The significance of the overlap, was assessed using the hypergeometric test.

For RNA-seq and ChIP-seq data analysis of embryo material, we used some additional procedures, as follows. Cufflinks and HTSeq: genes on the chromosome X and Y were filtered out from the FPKM values and the counts, respectively. Differential gene expression: Differentially expressed genes were identified using NOISeqBio package (version 2.8.0) (ref. 24) with default parameters and upper quartile normalization method. We used a threshold of 0.95 (the posterior probability of being differentially expressed).

To integrate H3K4me1-ChIP-seq data and RNA-seq data, we used the Epigenomix package (version 1.6.0), based on a Bayesian mixture model^23^. The Cuffdiff method was used to estimate FPKM values over all expressed protein coding genes. ChIP-seq values were assigned to each gene by counting the number of reads (extended to 300 bps), falling within a region of 3,000 bps around to TSS. A quantile normalization was applied to the ChIP-seq data and a correlation score Z between ChIP-seq data and RNA-seq data was calculated. Then the mixture Bayesian model was fitted to classify genes in ‘change' and ‘no change' groups.

For statistical significance analyses, we used hypergeometric tests, Fisher's exact test, binomial test and bootstrap approach. For the bootstrap approach we annotated randomly generated regions using ChIPpeakAnno estimating the number of genes associated to those regions. The process was reiterated 1,000 times.

For gene pathway analyses, we used InnateDB using the over-representation analysis option that scans overrepresentation of pathway genes, using Hypergeometric algorithm and BH correction. Pathways annotations from KEGG public databases significantly regulated with a *P* value<0.05 were reported.

To perform principal component analysis, we downloaded raw sequence of RNA-seq data of ESC, mesodermal and cardiac progenitors^18^ from https://b2b.hci.utah.edu/gnomex/. Both available biological replicates were used for the analysis. Similarly to our NT SiRNA (P19Cl6) RNA-seq data, we aligned the downloaded data to the mouse genome using TopHat with the default parameters, using annotation as guide. Then, we estimated the gene espression level with Cufflinks. We selected 13,840 genes expressed in P19Cl6 cells and in the downloaded data sets, and we normalized their expression value using full-quantile approach. Finally we computed the principal component analysis (in log-scale), selecting the first two components.

To evaluate H3K27Ac enrichment, we sequenced two ChIP-seq biological replicates and two input samples, which were pooled. Of a paired-end sequence data set, we selected only the R1 mate for the rest of the analysis. We aligned the sequences using Bowtie. As for the other samples described above, we kept only uniquely mapped reads and we filtered-out duplicates of PCR. Peaks were called using SICER and 20,308 were retained as present in both replicates. We annotated such regions with respect to TSS of protein coding genes, using ChipPeakAnno, obtaining 10,566 associated genes. We considered their FPKM in NT SiRNA. Forthermore, we selected 4,757 peaks enriched for both H3K27Ac and H3K4me1, obtaining a subset of 3,913 associated genes. Using Wilcoxon rank sum test (one-side), we compared their expression values with those 1,758 genes associated to the 1,699 peaks enriched for both Tbx1 and H3K4me1.

### Data availability

Sequence data that support the findings of this study have been deposited to the GEO database with the accession code GSE79941. The authors confirm that all other data appears in the article and Supplementary Information.

## Additional information

**Accession codes**: ChIP-seq and RNA-seq data are deposited in the Gene Expression Omnibus database under accession code GSE79941.

**How to cite this article**: Fulcoli, F. G. *et al*. Rebalancing gene haploinsufficiency *in vivo* by targeting chromatin. *Nat. Commun.* 7:11688 doi: 10.1038/ncomms11688 (2016).

## Supplementary Material

Supplementary Information Supplementary Figures 1-10 and Supplementary Tables 1-2

Supplementary Data 1 Expressed Genes

Supplementary Data 2 Tbx1 Golden Peaks

Supplementary Data 3 Differentially expressed Genes Tbx1 SiRNA vs NT SiRNA

Supplementary Data 4 Differentially methylated regions Tbx1 SiRNA+TCP vs Tbx1 SiRNA

Supplementary Data 5 Histone 3 Lysine 4 methyltransferases and Histone 3 Lysine 4 demethylases

Supplementary Data 6 Differentially expressed Genes Tbx1 SiRNA+TCP vs Tbx1 SiRNA

Supplementary Data 7 List of 608 rescued genes

Supplementary Data 8 Differentially methylated regions Tbx1 SiRNA+TCP vs Tbx1 SiRNA

Supplementary Data 9 Genes expressed in response to Tbx1 haploinsufficiency and to TCP

Supplementary Data 10 Changed Genes Mixture Model Tbx1^+/-^ vs WT

Supplementary Data 11 Differentially expressed Genes Tbx1^+/-^ vs WT

Supplementary Data 12 Differentially expressed Genes Tbx1^+/-^ vs WT (TCP rescued)

Supplementary Data 13 Primers used for Gene Expression analysis

Peer Review File
