# Supplementary material for: Rebalancing gene haploinsufficiency in vivo by targeting chromatin
Source: Nat Commun. 2016 Jun 3;7:11688. doi: 10.1038/ncomms11688 (PMC4895808; doi:10.1038/ncomms11688)
Supplement: Supplementary Information — Supplementary Figures 1-10 and Supplementary Tables 1-2 [file ncomms11688-s1.pdf]

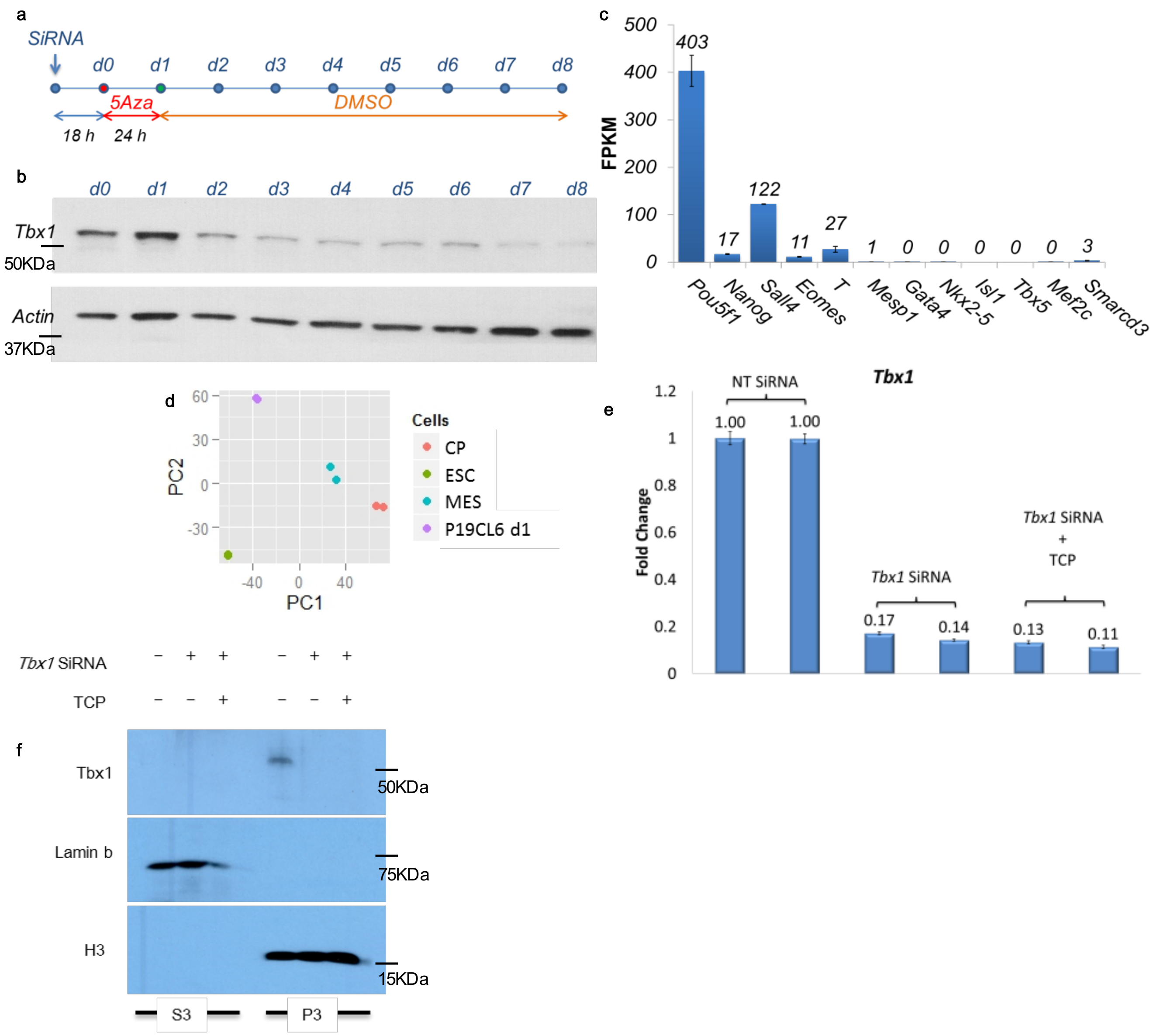

Supplementary Figure 1 | Experimental cell culture model used in this study. (a) Scheme of the P19Cl6 cell differentiation protocol. (b) Western blot showing the expression of the Tbx1 protein. The peak is at day 1, which is when we performed ChIP-seq and RNA-seq assays. (c) Chart depicting the expression levels, derived from 2 biological replicates of RNA-seq assays and expressed in FPKM of selected markers at day 1. A list of all the expressed genes at this stage is shown in the Supplementary Information Tab. 1. (d) Principal component analysis of the transcriptional profiles of three stages of cardiomyocyte differentiation of mouse embryonic stem cells: undifferentiated embryonic stem cells (ESC), cells expressing mesodermal markers (MES), Cardiac Precursors (CP) and P19Cl6 d1 cells. Data refer to two biological replicates per cell type. (e) Relative mRNA levels of *Tbx1* evaluated by quantitative real-time PCR in P19Cl6 cells transfected with non-targeting siRNA (NT) or *Tbx1* siRNA with or without TCP treatment. The data are represented as the mean±s.d.of 2 biological replicates. (f) Lysates of P19Cl6 cells transfected with non-targeting siRNA or *Tbx1* siRNA with or without TCP treatment were fractionated to obtain insoluble chromatin-enriched fraction (P3) and soluble nuclear fraction (S3). The distribution of Tbx1, Lamin b and H3 was detected by western blotting. Note the association of Tbx1 with chromatin. TCP: Tranylcypromine.

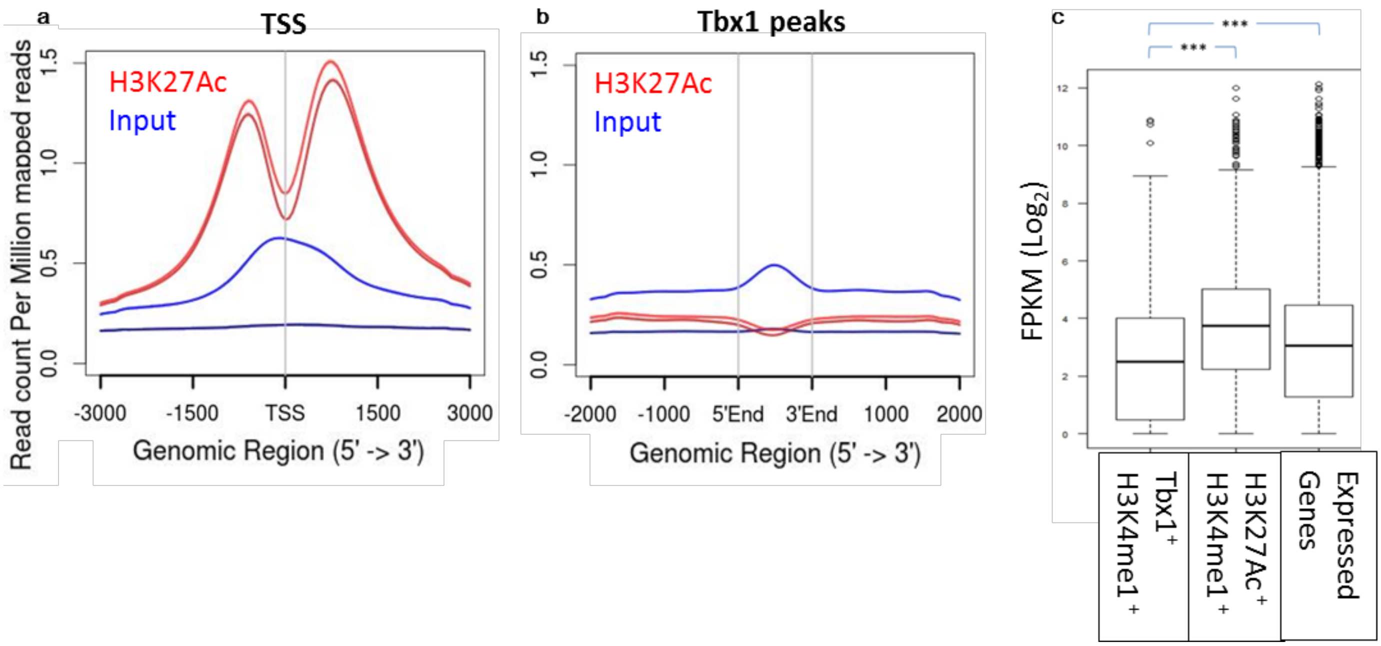

Supplementary Figure 2 | Analysis of H3K27Ac enrichment, ChIP-seq data. (a) Map of H3K27Ac enrichment relative to TSS and (b) H3K27Ac occupancy relative to Tbx1 peaks in two biological replicates. 5' and 3' ends refer to the extremities of the peak. Note the absence of H3K27Ac localization in Tbx1 peak regions. The graphs show ChIP-seq data from two independent experiments. (c) Boxplot showing the expression distribution of genes associated with regions positive for Tbx1 and H3k4me1, genes associated with regions positive for H3k4me1 and H3K27Ac, and all expressed genes. Genes associated with Tbx1+;H3K4me1+ regions are significantly less expressed than any of the other two categories. *** P<0.001.

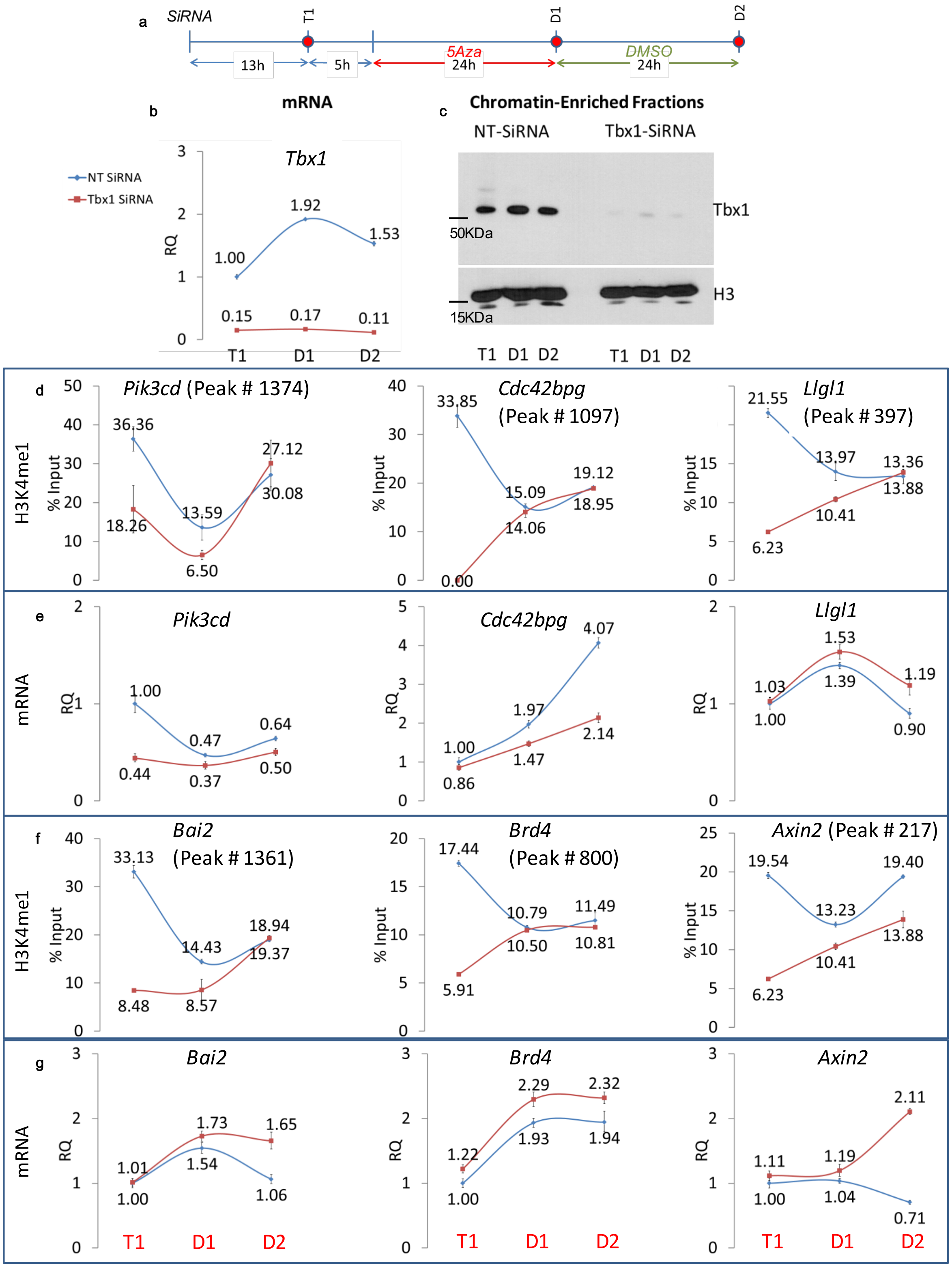

Supplementary Figure 3 | Time-course analysis. (a) Scheme of time-course protocol: P19Cl6 cells are transfected with Tbx1 SiRNA or Non-targeting SiRNA. The first point of analysis (T1) is at 13 hours post transfection; the second and third point (D1 and D2) are at 24 hours and 48 hours post induction with 5-aza respectively. (b) Q-RT-PCR analysis for *Tbx1* gene expression. (c) Western analysis of chromatin-enriched fractions with an anti-Tbx1 antibody. An anti-H3 antibody was used for normalization. (d, f) Time-course Q-Chip assays with an anti-H3K4me1 antibody for a group of Tbx1 binding-sites (Peaks IDs and associated genes are indicated as in Supplementary Information Tab.2). (e, g) Time-course Q-RT-PCR assays for the group of genes associated to the selected Tbx1 binding-sites. The data are represented as the mean±s.d.of 2 biological replicates.

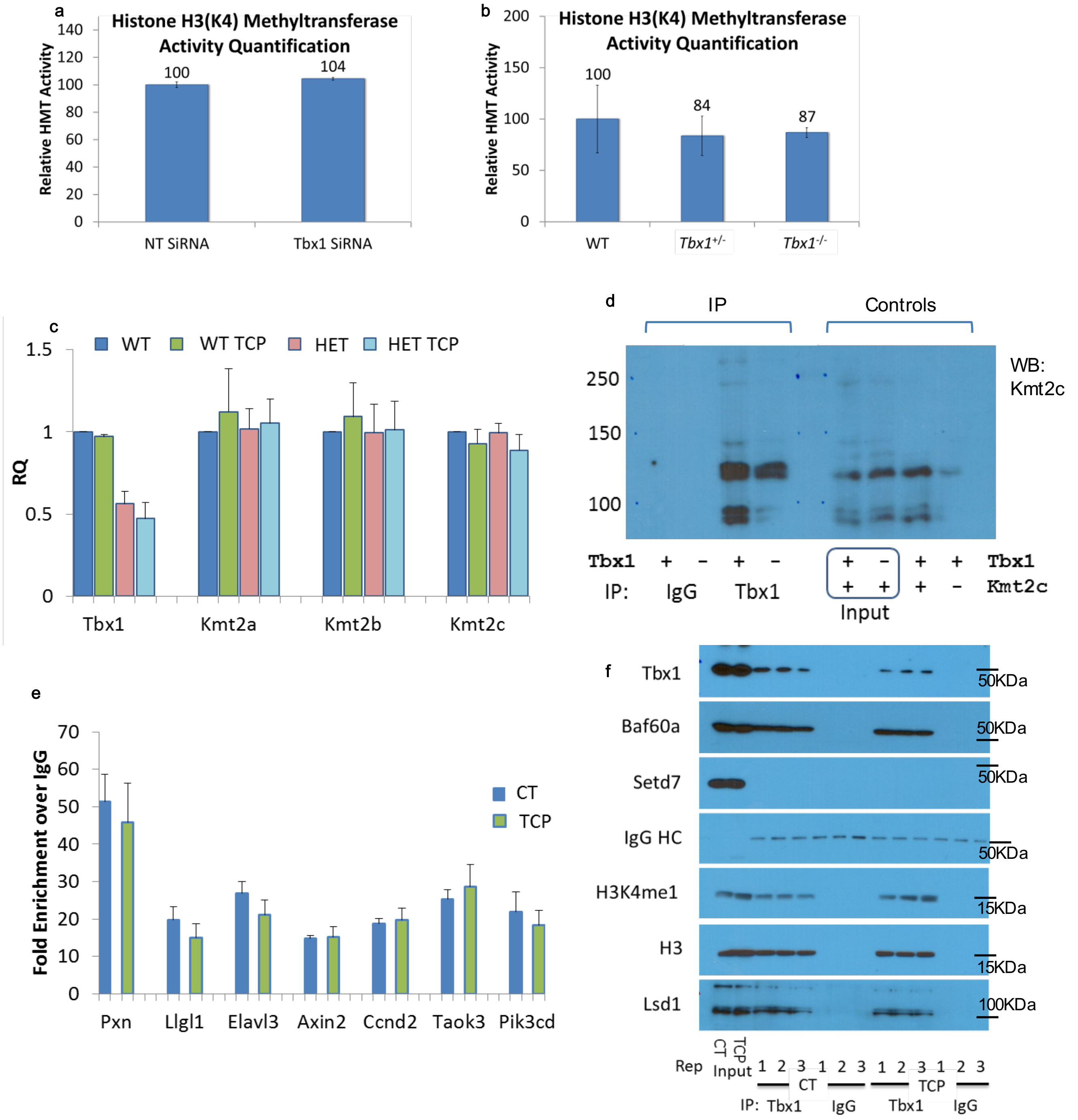

Supplementary Figure 4 | Tbx1, H3K4 methylation and interactions with methyltransferases. (a) Relative HMTase activity of nuclear extracts of P19Cl6 cells transfected with non-targeting siRNA or Tbx1 siRNA were measured using colorimetric quantification (See Materials and Methods). HMTase assays were done in duplicate individual experiments and all samples were in duplicate. (b) Relative HMTase activities of nuclear extracts of WT, *Tbx1*$^{+/-}$ and *Tbx1*$^{-/-}$ E9.5 embryos. HMTase assays were done in at least 4 biological replicates. The error bars indicate standard deviations. (c) Expression analysis of *Kmt2a*, *Kmt2b* and *Kmt2c* in WT and *Tbx1*$^{Lacz/+}$ E9.5 embryos with and without TCP treatment. 3 biological replicates were considered for analysis. The error bars indicate s.d. (d) Western blot analysis of co-immunoprecipitation experiments with an anti-Tbx1 antibody or rabbit IgG (control) and nuclear extracts of P19Cl6 cells transfected with non-targeting siRNA or Tbx1 siRNA. *Kmt2c* siRNA was used as control for signal specificity. (e) Quantitative ChIP (q-ChIP) assay on a subset of Tbx1 binding sites in P19Cl6 cells with and without TCP treatment using an anti-Kmt2c antibody, followed by quantitative real-time PCR in 3 biological replicates. Error bars: s.d. (f) ChIP–Western blot analyses of three independent IP experiments (1, 2, and 3) with endogenous proteins from P19Cl6 differentiating cells with or without TCP treatment. Immunoprecipitation was carried out using an anti-Tbx1 antibody or rabbit IgG (control).

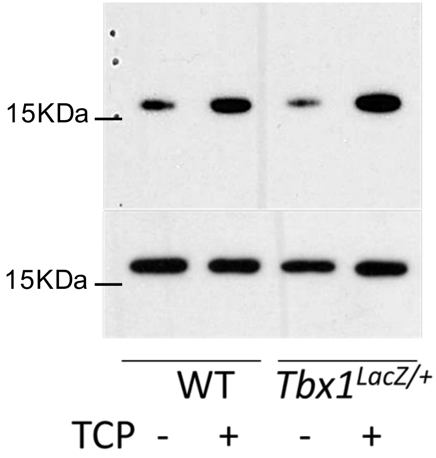

Supplementary Figure 5 | Western blotting analyses of histones isolated from WT or *Tbx1*$^{Lacz/+}$ E9.5 embryos untreated or TCP-treated. H3K4me1 and H3 were detected with specific antibodies.

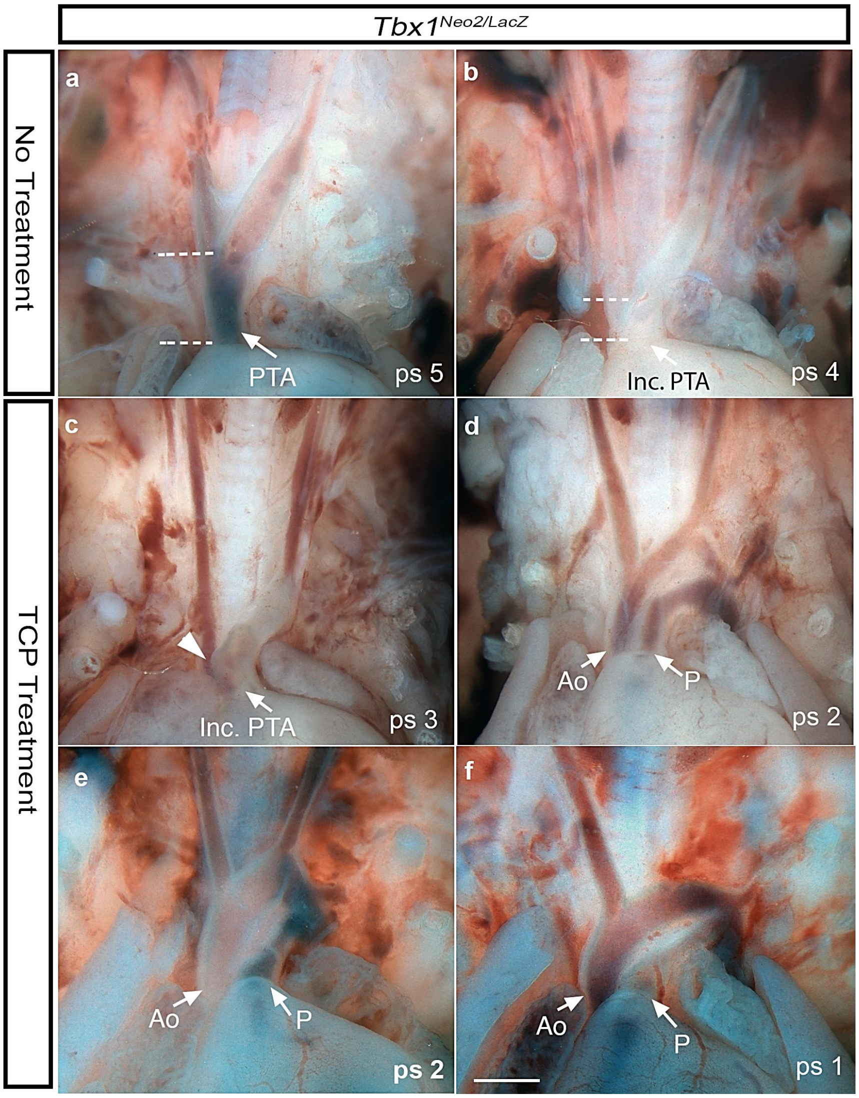

Supplementary Figure 6 | External views of cardiac outflow tract (OFT) phenotype E18.5 *Tbx1*$^{Neo2/LacZ}$ embryos with and without TCP treatment. (a-b) Complete and incomplete persistent truncus arteriosus (PTA) seen in *Tbx1*$^{Neo2/LacZ}$ mutants without TCP treatment. (a) the OFT is undivided and is classified as phenotypic score (ps) 5. The unseptated truncal region is delimited by dashed lines. (b) The OFT is unsptated only in the proximal region and is classified as ps 4. (c-f) TCP treated *Tbx1*$^{Neo2/LacZ}$ embryos. (c) Incomplete PTA features a further but unevenly septated OFT and classified as ps 3. (d-e) In these samples, the great arteries are completely separated and were diagnosed as double outlet right ventricle (DORV) after histological analyses (see Fig. 4). Note the great arteries side-by-side in sample d, while they are more correctly positioned in sample e. (f) normal OFT morphology, classified as ps 1. Ao, aorta; P, pulmonary trunk. Scale bar is 500 μm.

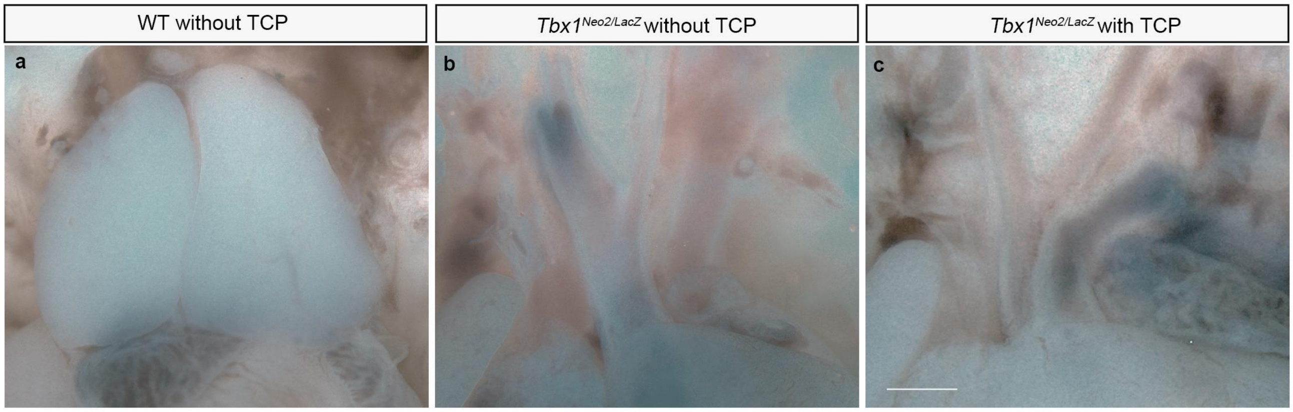

Supplementary Figure 7 | Thymic organ development is not affected by TCP. (a) E18.5 WT embryo with normal development of thymus. (b-c) *Tbx1*$^{Neo2/LacZ}$ embryos with aplasia of the thymus, irrespective of TCP treatment. Scale bar is 500 μm.

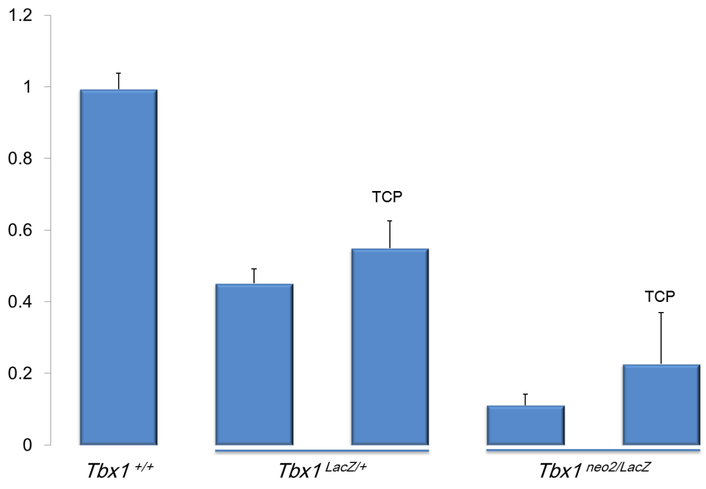

Supplementary Figure 8 | TCP treatment of embryos has no significant effect on Tbx1 gene expression. qRT-PCR of RNA from *Tbx1*$^{Neo2/+}$ and *Tbx1*$^{Neo2/LacZ}$ E9.5 embryos, with and without TCP treatment. The small differences shown are not statistically significant. Error bars: s.e.m.; n=3; two-tailed Student's t-test.

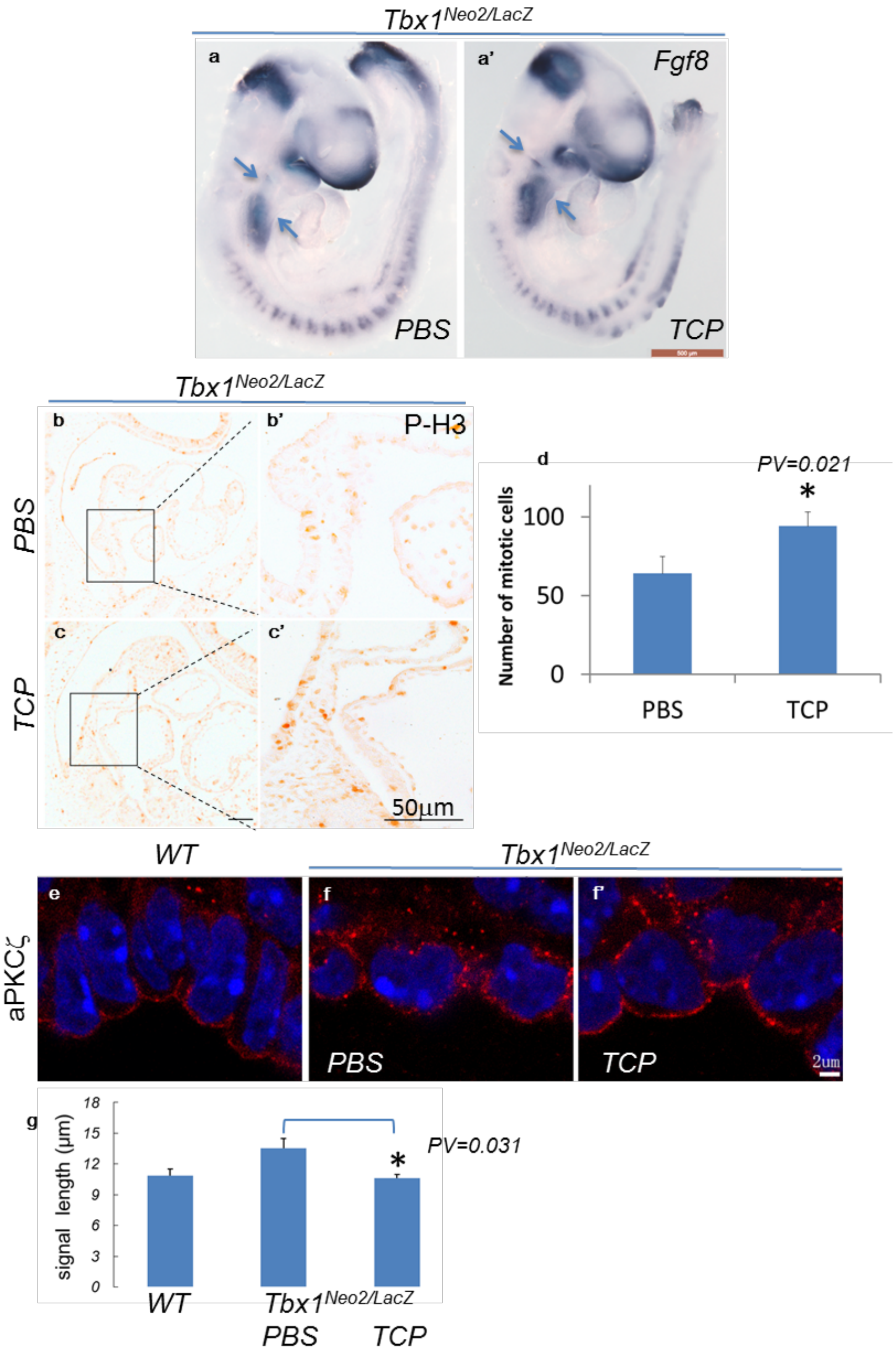

Supplementary Figure 9 | Effects of TCP treatment on E9.5 embryos. Pregnant females were injected with TCP at E7.5, E8.5, and E9.5. (a-a') whole-mount in situ hybridization with *Fgf8*. *Fgf8* expression is regulated by Tbx1 but is not a direct target, according to our ChIP-seq data. Arrows indicate the regions of expression enhancement/expansion in the TCP-treated embryo. The scale bar is 500µm. (b-d) Immunohistochemistry using an anti P-H3 antibody to reveal mitotic cells in the second heart field region of PBS and TCP-treated E9.5 embryos (b-c'). Quantitative data are summarized on the histogram (d) and show significant enhancement of mitotic cells in TCP treated embryos (data from 3, somite-matched embryos per experimental point). (e-g) Immunofluorescence using an anti aPKCζ antibody on the second heart field region (anterior dorsal pericardial wall) of E9.5 embryos (transverse sections). In WT embryos, the signal is at the apical region of the epithelial layer of the second heart field (e). In $Tbx1^{Neo2/LacZ}$ embryos, the signal is stronger and tends to surround cells of the epithelial layer (f). in treated $Tbx1^{Neo2/LacZ}$ embryos, the signal morphology is more similar to that of WT embryos (f'). The histogram (g) summarizes measurements of membrane signal length and shows significant reduction in TCP-treatment embryos (data from three somite-matched embryos per experimental point). Error bars: s.e.m.; n=3; two-tailed Student's t-test.

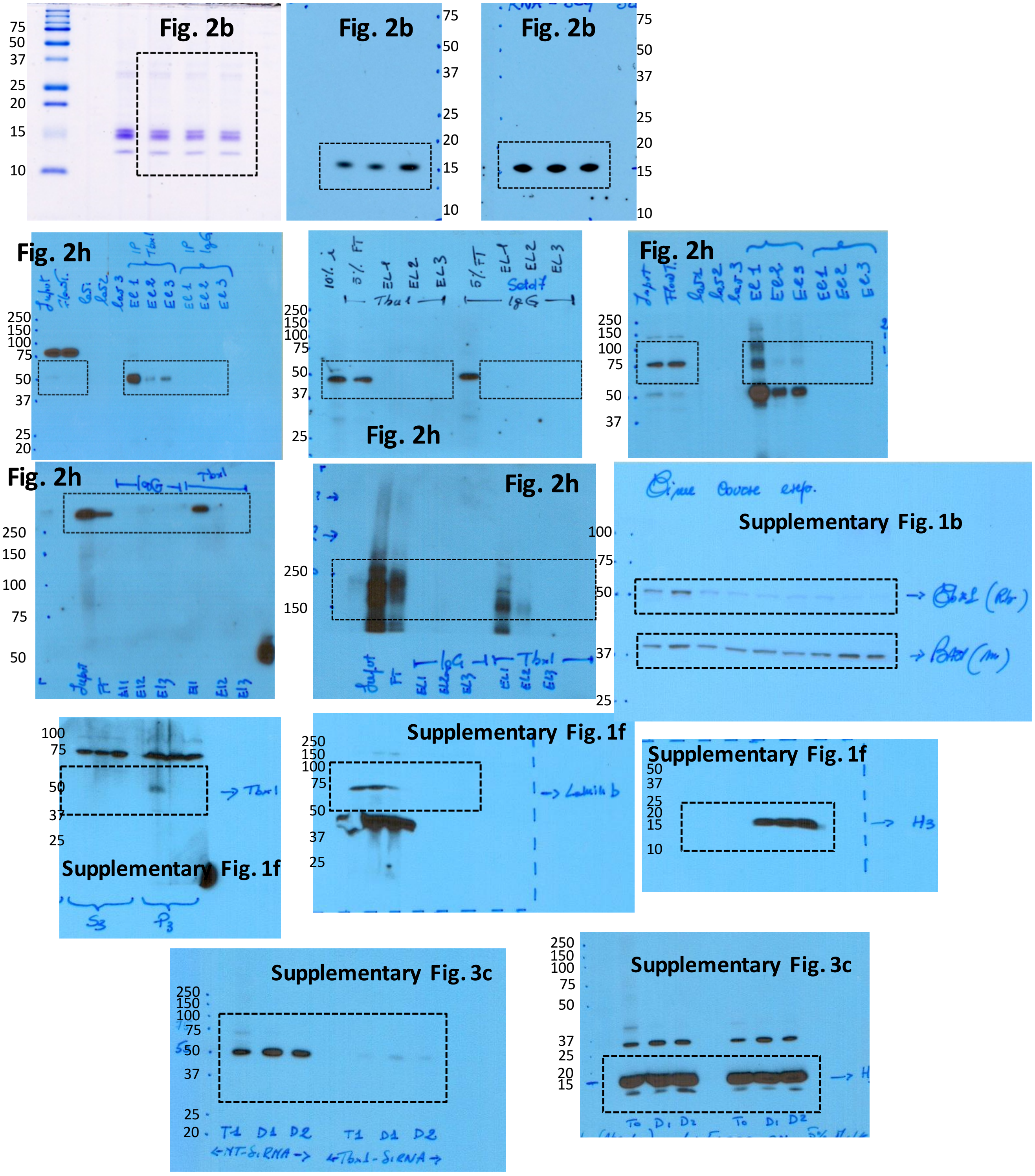

Supplementary Figure 10 | Uncropped Scans of autoradiographies shown in Fig. 2 and Supplementary Figs. 1-3.

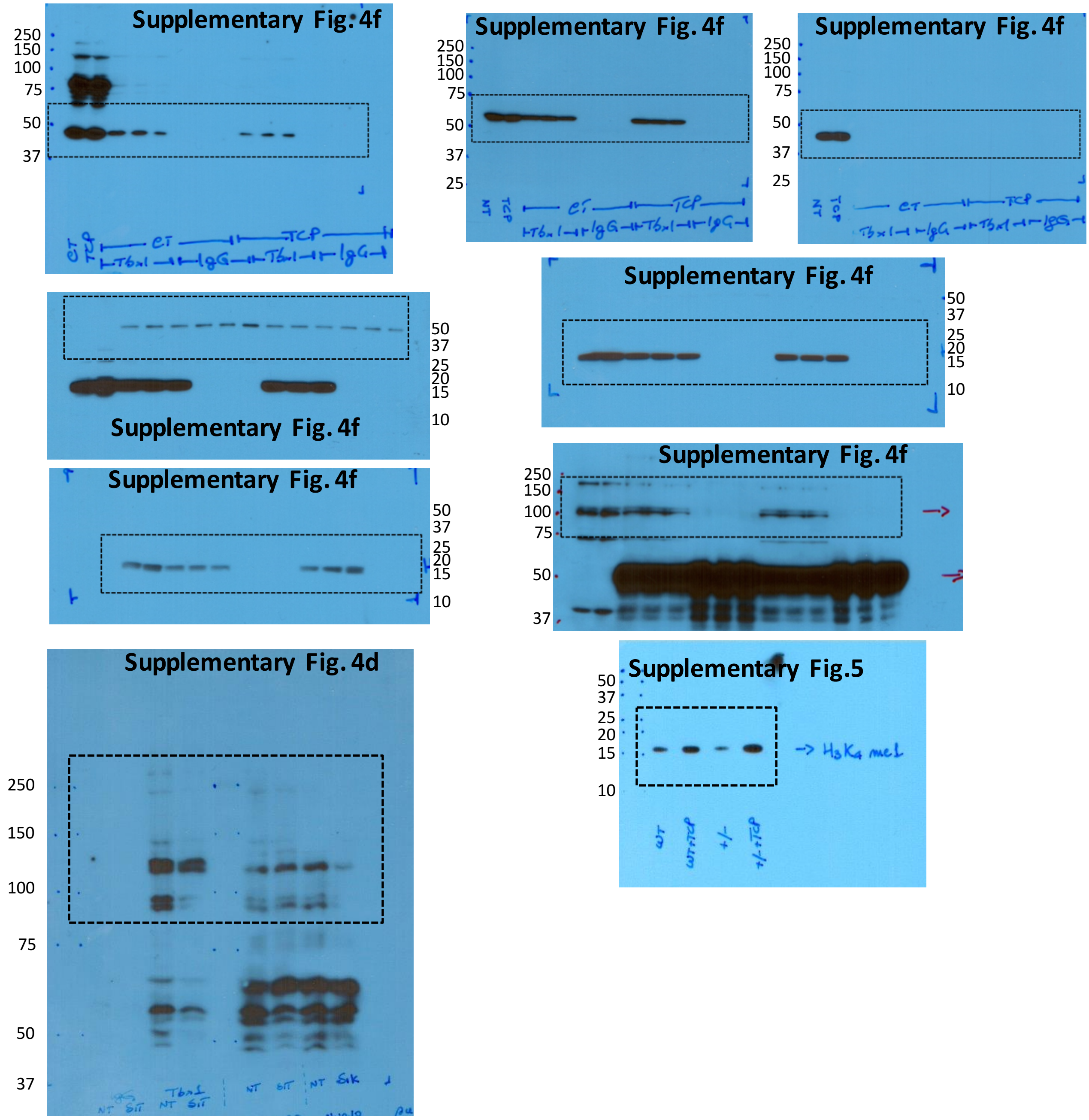

Supplementary Figure 10 (Continued) | Uncropped Scans of autoradiographies shown in Supplementary Figs. 4-5.

	Non Targeting	*Tbx1* SiRNA	*Lsd1* SiRNA	*Lsd2* SiRNA	*Tbx1-Lsd1* SiRNA	*Tbx1-Lsd2* SiRNA	*Tbx1-Lsd1-Lsd2* SiRNA	Rescue Lsd1 KD	Rescue Lsd2 KD
Als2cl	1.00	0.79	0.93	1.69	1.24	1.25	1.92	Y	Y
Pxn	1.00	0.84	1.01	1.48	0.94	1.26	1.44	Y	Y
Cdc42bpg	1.00	0.84	1.21	1.17	0.90	0.83	1.01	Y	N
Pik3cd	1.00	0.88	1.28	1.01	1.09	0.63	0.83	Y	N
Akt1	1.00	1.13	1.29	0.95	1.44	0.59	1.40	Y	N
Sufu	1.00	1.43	0.95	0.83	1.29	0.93	1.11	N	Y
Ppargc1b	1.00	1.48	1.04	1.69	0.97	2.40	1.51	Y	N
Llgl1	1.00	1.53	1.10	0.95	1.45	1.47	1.34	N	N
Bai2	1.00	1.88	0.96	0.85	1.49	1.07	0.99	N	Y
Elavl3	1.00	1.90	1.26	0.67	1.96	1.22	1.98	N	Y
Axin2	1.00	2.14	0.91	0.89	1.43	1.63	1.60	Y	Y
Ccnd2	1.00	3.17	0.73	1.23	1.24	2.29	1.96	Y	N

Tbx1	1.00	0.12	0.79	1.14	0.12	0.14	0.10
Lsd1	1.00	1.44	0.20	0.87	0.23	0.91	0.17
Lsd2	1.00	1.19	0.82	0.51	0.89	0.67	0.52

Supplementary Table 1 | Loss of *Lsd1* and/or *Lsd2* recapitulates the effects of TCP on a group of Tbx1 target genes.
Mean values of relative expression of a group of Tbx1 target genes rescued by TCP treatment, all experiments were performed in triplicate (raw data in Source data). The numerical values are embedded in a heat map with colors ranging from blue (for up-regulated genes) to red (for down-regulated genes), in white are indicated the Non-Targeting siRNA Controls. The top row indicates the experimental condition: SiRNA of *Tbx1*, *Lsd1* or *Lsd2* alone; combined SiRNA of *Tbx1* and *Lsd1* or *Tbx1* and *Lsd2*; combined SiRNA of *Tbx1*, *Lsd1* and *Lsd2*.

a

IVth Pharyngeal arch artery defects E10.5

Genotype	*Tranylcypromine*	**Normal**	**Affected**
Tbx1 $^{+/+}$	Untreated	45	0
	Treated	25	0
Tbx1 $^{LacZ/+}$	Untreated	6	17 (74%)
	Treated	14	8 (36%)
			**P‹0.001**

b

Cardiac outflow tract phenotype in Tbx1$^{Neo2/LacZ}$ embryos at E18.5

Treatment TCP	n	Normal *(ps 1)*	DORV *(ps 2)*	Partial PTA *(ps 3)*	Incomplete PTA *(ps 4)*	PTA *(ps 5)*	*Average phenotypic score*
No	6	0	0	0	2	4	4.7
Yes	9	1	4	3	1	0	2.4

Supplementary Table 2 | Effect of TCP on cardiovascular phenotype of *Tbx1*$^{LacZ/+}$ (a) and *Tbx1*$^{Neo2/LacZ}$ (b) embryos.
